# The Hippo pathway links adipocyte plasticity to adipose tissue fibrosis

**DOI:** 10.1038/s41467-022-33800-0

**Published:** 2022-10-13

**Authors:** Hongyu Shen, Xun Huang, Yiheng Zhao, Dongmei Wu, Kaili Xue, Jingfei Yao, Yushuang Wang, Nan Tang, Yifu Qiu

**Affiliations:** 1 Institute of Molecular Medicine, Beijing Key Laboratory of Cardiometabolic Molecular Medicine, College of Future Technology, Peking University, 100871 Beijing, China; 2 Peking-Tsinghua Center for Life Sciences, Peking University, 100871 Beijing, China; 3 Academy for Advanced Interdisciplinary Studies, Peking University, 100871 Beijing, China; 4 National Institute of Biological Sciences, 102206 Beijing, China; 5 Tsinghua Institute of Multidisciplinary Biomedical Research, Tsinghua University, 100084 Beijing, China

**Keywords:** Metabolic diseases, Growth factor signalling, HIPPO signalling, Extracellular matrix, Adipocytes

## Abstract

Fibrosis disrupts adipose tissue (AT) homeostasis and exacerbates metabolic dysfunction upon chronic caloric excess. The molecular mechanisms linking adipocyte plasticity to AT fibrosis are largely unknown. Here we show that the Hippo pathway is coupled with TGFβ signaling to orchestrate a cellular and/or functional shift of adipocytes from energy storage to extracellular matrix (ECM) remodeling in AT fibrosis. We found that *Lats1/2*-knockout adipocytes could dedifferentiate into DPP4^+^ progenitor cells and convert to DPP4^−^ myofibroblasts upon TGFβ stimulation. On the other hand, Hippo pathway inhibition during obesity impaired adipocyte identity while promoted ECM remodeling activity of adipocytes. Macrophages recruited by CCL2 produced TGFβ to accelerate AT fibrosis. YAP and TAZ, the Hippo downstream effectors, enhanced SMAD2 stability to promote fibrotic responses. Importantly, inhibition of YAP/TAZ activity in obese mice markedly relieved AT fibrosis and improved metabolic homeostasis. Together, our findings identify the Hippo pathway as a molecular switch in the initiation and development of AT fibrosis, implying it as a therapeutic target.

## Abstract (web summary)

Adipose tissue fibrosis is connected to obesity-related metabolic dysfunction. Qiu and colleagues discover that the Hippo pathway acts as a molecular switch in the initiation and development of adipose tissue fibrosis upon TGFβ stimulation.

## Introduction

Adipose tissue (AT) undergoes continuous remodeling for the adaptation to energy alterations during obesity^1,2^. Maladaptive AT remodeling is characterized by adipocyte hypertrophy (an increase in adipocyte size), inflammation and fibrosis, and associated with metabolic abnormalities like type 2 diabetes^3^. In the context of nutrient excess, AT expands through hypertrophy and hyperplasia (an increase in adipocyte number through de novo differentiation). Hypertrophic adipocytes exhibit reduced expression of fat identity genes that leads to an impaired capacity to store excess lipid, and release inflammatory adipokines to aggravate AT microenvironment^4^. The microenvironment, comprised of fibroblasts, immune cells and endothelial cells, is implicated in the progression of AT fibrosis that is defined as an excessive accumulation of extracellular matrix (ECM) and impairs AT function^3^. Adipocyte-specific overexpression of endotrophin, the cleavage C5 domain of the COL6α3, aggravates high-fat-diet (HFD)-induced fibrosis and metabolic dysfunction^5^. Importantly, AT fibrosis is associated with insulin resistance of individuals with obesity^6–8^.

AT fibrosis is regulated by hypoxia, PRDM16, and PDGF signaling. Hypoxia-inducible factor 1α, a key transcriptional factor induced by hypoxia, triggers AT fibrosis by inducing transcription of ECM components and changing cellular redox status to influence collagen crosslinking enzymes like lysyl oxidase (LOX)^9^. PRDM16 forms a complex with GTF2IRD1 in white adipocytes upon adrenergic stimulation like cold exposure and CL-316,243 treatment to reduce AT fibrosis, or elicits an adipocyte-to-precursor paracrine signal that act on precursor cells to block myofibroblast differentiation^10,11^. Activation of PDGFRα signaling in perivascular cells drive AT fibrosis^12^. During HFD-induced obesity, PDGFRα^+^CD9^hi^ progenitors express ECM to promote AT fibrosis, whereas PDGFRα^+^CD9^lo^ progenitors are prone to undergo adipogenesis^13^. A recent study identified a subpopulation of mature adipocytes that exhibited reduced fat cell identity and functionally shifted to an ECM-remodeling status in response to HFD-induced obesity^14^. However, whether and how adipocyte plasticity contributes to AT fibrosis remain poorly understood.

Transforming growth factor β (TGFβ) is the master regulator of fibrosis, which activates fibroblasts and myofibroblasts and accelerates ECM accumulation. TGFβ includes three isoforms (TGFβ1, TGFβ2, TGFβ3) in mammals. In canonical TGFβ signaling, TGFβ binds to TGFβ receptor 2 (TGFβR2), which then recruits TGFβ receptor 1 (TGFβR1) to enhance phosphorylation of SMAD2 and SMAD3. Phosphorylated SMAD2/3 form a complex with SMAD4 to translocate to the nucleus and induce ECM gene expression^15^. In addition, TGFβ can signal through non-canonical pathways mediated by MAP kinases (ERK, JNK, p38), PI3K/AKT, and Rho-associated coiled-coil containing protein kinases (ROCKs) to promote fibrosis^16^.

The Hippo pathway plays a remarkable role in the regulation of organ size and tissue homeostasis. Its core components comprise a kinase cascade and a transcriptional module. Activation of the Hippo pathway kinases LATS1/2 phosphorylates YAP/TAZ (the downstream transcriptional coactivators) and promotes their cytoplasmic retention and proteolytic degradation. In addition, mechanical cues can directly regulate YAP/TAZ activation via relieving their sequestration by nuclear ARID1A-SWI/SNF complex^17–19^. Non-phosphorylated YAP/TAZ shuttle into the nucleus and bind to the transcription factors, particularly TEADs, to drive target gene expression^20^. YAP and TAZ activity is critical for PKA-induced adipogenesis^21^. TAZ controls a cell fate choice between adipogenesis and osteogenesis of mesenchymal stem cells by interaction with PPARγ or Runx2^22^. Although the Hippo pathway has been implicated in the development of adipocyte in vitro, whether it regulates adipocyte plasticity in vivo in the progression of AT fibrosis was unclear.

In this study, we reveal that the Hippo pathway links adipocyte plasticity to AT fibrosis via close coordination with TGFβ signaling. Using genetic mouse models, we uncover the cellular and molecular mechanisms controlling AT fibrosis development. These mechanisms also apply to obesity-induced AT fibrosis. Furthermore, our genetic and chemical genetic data suggest that targeting the YAP/TAZ–TEADs axis could largely ameliorate AT fibrosis.

## Results

### Obesity is associated with Hippo pathway inhibition and fibrotic induction in ATs

**Fig. 1:**
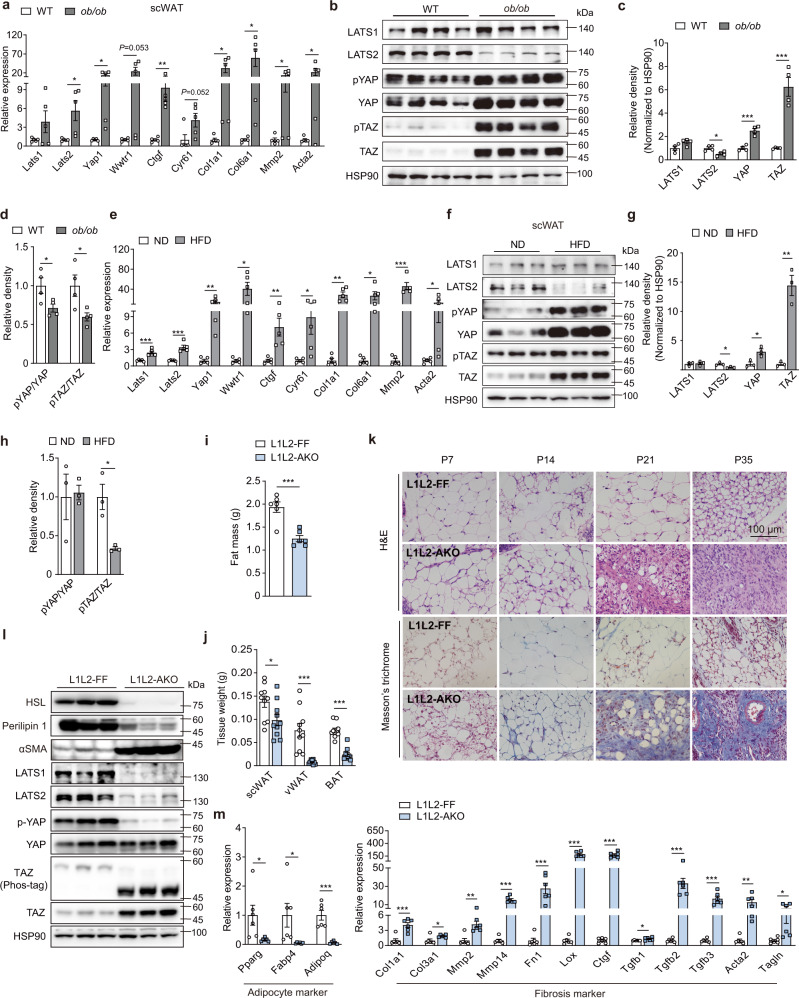
*Lats1/2* deficiency leads to fat remodeling. **a**–**d** Male WT or *ob/ob* mice were analyzed at 12 weeks old. **a**, Real-time quantitative PCR (RT-qPCR) analysis of genes involved in Hippo pathway and fibrotic response in scWAT (*n* = 5 mice). **b**, Immunoblot analysis of LATS1/2, YAP/TAZ, YAP phosphorylated at Ser112 (p-YAP) and TAZ phosphorylated at Ser89 (p-TAZ) in scWAT. **c** Quantification of protein expression from scWAT shown in **b** (*n* = 4 mice). **d** Quantification of phosphorylation levels of YAP/TAZ (*n* = 4 mice). **e**–**h** Male WT mice were fed a HFD or ND for 18 weeks. **e** RT-qPCR analysis of genes involved in Hippo pathway and fibrotic response in scWAT (*n* = 5 mice). **f** Immunoblot analysis of LATS1/2, YAP/TAZ, p-YAP and p-TAZ in scWAT. **g** Quantification of protein expression from scWAT shown in **f** (*n* = 3 mice). **h** Quantification of phosphorylation levels of YAP/TAZ (*n* = 3 mice). **i**–**m** Male *Lats1*^*f/f*^*Lats2*^*f/f*^ (L1L2-FF) and *Lats1*^*f/f*^*Lats2*^*f/f*^ *Adipoq*^*Cre*^ (L1L2-AKO) mice were analyzed at 5 weeks old. **i** Quantification of body fat mass by DXA (*n* = 6 mice). **j** Quantification of scWAT, vWAT and BAT weight (*n* = 10 mice). **k** Representative sections of scWAT with H&E staining or Masson’s trichrome staining at the indicated age. **l** Immunoblot analysis of adipocyte identity and fibrotic protein expression. **m** RT-qPCR for adipocyte and fibrotic marker gene expression in scWAT (*n* = 6 mice). Data are means ± SEM. Two-tailed unpaired Student’s *t* test; *P < 0.05, **P < 0.01, ***P < 0.001. Exact *P* values are provided in a Source data file.

To investigate the role of the Hippo pathway in obesity-induced AT fibrosis, we first profiled the expression of several pathway components in the hyperphagic *Leptin*-deficient *ob/ob* mouse model, which has been shown to exhibit elevated ECM deposition in the obese state^9^. Obesity robustly induced transcription of fibrotic genes in both subcutaneous white AT (scWAT) and visceral white AT (vWAT), including *Col1a1*, *Col6a1*, and *Mmp2* (Fig. 1a and Supplementary Fig. 1a), consistent with RNAseq analysis of adipocytes that acquire a fibroblast-like transcriptional signature in response to a HFD feeding^23^. Meanwhile, obesity increased messenger RNA (mRNA) level of Hippo pathway effectors *Yap1*, *Wwtr1 (Taz)*, and their downstream targets, such as *Ctgf* and *Cyr61* (Fig. 1a and Supplementary Fig. 1a). Transcriptome analysis of human abdominal scWAT also revealed that obesity induces the expression of genes of the fibrosis pathway and YAP conserved signatures (Supplementary Fig. 2a, b). *Lats1* and *Lats2* mRNA were upregulated in both obesity models (Fig. 1a, e and Supplementary Fig. 1a, k). At the protein level, LATS2 was markedly reduced whereas YAP/TAZ was significantly upregulated both in scWAT (Fig. 1b, c) and vWAT (Supplementary Fig. 1b, c) of obese mice compared to lean controls. This may imply a compensatory mechanism by which *Lats1* and *Lats2* mRNA expression is induced. The relative amount of phosphorylated YAP and TAZ (pYAP and pTAZ) was reduced, indicating inhibition of the Hippo pathway (Fig. 1d and Supplementary Fig. 1d). The WATs of female mice exhibited similar expression patterns of Hippo pathway components and fibrotic genes (Supplementary Fig. 1e–j). In addition, the Hippo pathway was inhibited and the expression of YAP/TAZ and their downstream targets was increased in another obese mouse model, which was HFD-induced obesity, accompanied by a robust induction of fibrotic program (Fig. 1e–h and Supplementary Fig. 1k–m). We also found that *WWTR1* (encoding *TAZ*) and *YAP1* mRNA expression are positively correlated with the AT fibrosis levels of individuals, as marked by normalized expression values for COL6A3 and MMP2 (Supplementary Fig. 2c–f). Together, these findings reveal a strong positive correlation between Hippo pathway inactivation and fibrotic induction during obesity-induced AT fibrosis.

### Hippo pathway inactivation in adipocytes leads to a substantial AT remodeling

To assess the function of Hippo pathway in ATs, we crossed *Lats1*^*f/f*^*Lats2*^*f/f*^ (L1L2-FF) with *Adipoq*^*Cre*^ mice to generate adipocyte-specific *Lats1/2* double-knockout mice, named *Lats1*^*f/f*^*Lats2*^*f/f*^*Adipoq*^*Cre*^ (L1L2-AKO). *Lats1/2* deletion results in a reduction of *Lats1*/*2* mRNA expression in scWAT and brown AT (BAT) but not in non-ATs, indicating a selective deletion of *Lats1/2* in ATs (Supplementary Fig. 3a, b). Quantification of mRNA in adipocytes isolated from P7 L1L2-FF and L1L2-AKO male mice showed ∼70% reduction of *Lats1* and *Lats2* expression in scWAT (Supplementary Fig. 3c). Compared to L1L2-FF controls, L1L2-AKO mice exhibited reduced body size and had less body weight at the age of five weeks (Supplementary Fig. 3d, e). They started to die from postnatal day 28 (P28) and none survived past P42 (Supplementary Fig. 3f). Adipocyte *Lats1/2* deletion resulted in smaller liver but indistinguishable hepatocyte morphology (Supplementary Fig. 3g). Accordingly, L1L2-AKO mice had less fat mass and tissue weight of scWAT, vWAT and BAT (Fig. 1i, j and Supplementary Fig. 3h), which was in accordance with a decrease of plasma non-esterified fatty acids (NEFAs) (Supplementary Fig. 3i). Concomitantly, *Lats1/2*-deficient scWAT exhibited a massive loss of fat compared to that of their L1L2-FF littermates, as evidenced by a pronounced reduction in mRNA expression of adipocyte marker genes like *Pparg*, *Fabp4*, and *Adipoq* and in protein expression of HSL and Perilipin 1, two key lipolytic enzymes (Fig. 1l, m). These results indicate that adipocyte *Lats1/2* deletion leads to a fat-loss phenotype. This fat-loss phenotype was not attributed to an apoptosis of adipocytes as no cleaved caspase 3 was observed in L1L2-AKO mice (Supplementary Fig. 3j).

Meanwhile, L1L2-AKO scWAT showed a drastic increase in the expression of fibrotic genes including *Col1a1* and *Col3a1* (responsible for collagen production), *Mmp2* and *Mmp14* (collagen breakdown), *Fn1* (matrix assembly), *Lox* (matrix cross-linking) and *Tagln* (myofibroblast) (Fig. 1m), indicating an accumulation of excessive ECM upon adipocyte *Lats1/2* absence. Additionally, *Acta2*/αSMA expression was robustly upregulated at both mRNA and protein levels, implying that myofibroblasts were recruited and activated to promote fibrosis in AT (Fig. 1l, m). We isolated adipocytes from P7 L1L2-AKO scWAT to perform RNAseq analysis and found that *Lats1/2*-deficient adipocytes showed an increase of genes involved in ECM remodeling (Supplementary Fig. 3l). Next, we confirmed the fat-loss and fibrosis phenotype of L1L2-AKO mice by staining approaches: the hematoxylin and eosin (H&E) staining showed that lipid-enriched adipocytes gradually disappeared in the scWAT depots of the knockout mice during growth (Fig. 1k), whereas Masson’s trichrome staining showed that collagen deposition gradually increased and reached a maximum at five weeks old (Fig. 1k). Picrosirius red staining provided another evidence to confirm the abnormal accumulation of collagens (Supplementary Fig. 3k). *Lats1* and *Lats2* play a redundant role in maintaining AT homeostasis as revealed by that single knock-out of *Lats1* (L1-AKO) or *Lats2* (L2-AKO) does not lead to fat loss or induce AT fibrosis (Supplementary Fig. 3o–q), which is consistent with what was reported in other tissues^24,25^. We observed a similar body size and body weight of L1L2-AKO mice compared to L1L2-FF mice at P21 when AT fibrosis was robustly induced (Fig. 1k and Supplementary Fig. 3m, n), excluding a potential indirect effect of reduced body side on AT biology of L1L2-AKO mice at P35. Collectively, these findings demonstrate that inactivation of *Lats1/2* in adipocyte is sufficient to induce drastic fat remodeling, suggesting that the Hippo pathway may function as a key molecular switch to maintain AT homeostasis.

### *Lats1/2* deficiency-induced AT fibrosis does not depend on MST1/2

MST1 was downregulated while MST2 not altered in diet-induced obesity model and both MST1 and MST2 were unchanged in genetic *ob/ob* model (Supplementary Fig. 4a, b), implying that the Hippo pathway was partially inactivated in obesity. We further generated *Mst1*^*f/f*^*Mst2*^*f/f*^*Adipoq*^*Cre*^ (M1M2-AKO) mice to specifically delete *Mst1/2* in adipocytes. We found no change of adipocyte identity and AT fibrosis at 5 weeks old, not like what was observed with L1L2-AKO mice (Supplementary Fig. 4c–f). This finding indicates that *Lats1/2* deficiency-induced AT fibrosis does not depend on their canonical upstream kinases MST1/2.

### Hippo pathway inactivation alone is not sufficient to induce AT fibrosis

To determine the cell-autonomous role of LATS1/2 in AT remodeling, we depleted *Lats1/2* in differentiated adipocytes using CRISPR-Cas9 approach to mimic the in vivo state of L1L2-AKO mice. We infected *Cas9*^*Tg/+*^ stromal vascular fraction (SVF) with AAV carrying guide RNAs (gRNAs) against *Lats1/2* on day 2 post induction to effectively knockout LATS1/2 in mature adipocytes by day 6 (Supplementary Fig. 5a, b). Two independent gRNAs were able to effectively knockout *Lats1/2* (Supplementary Fig. 5e) and impaired adipocyte identity in a knockout efficiency-dependent manner, with no induction of the expression of fibrotic program (Supplementary Fig. 5c, d). We thus chose the more effective gRNA1 for the following experiments. Together, these results suggest that LATS1/2 inactivation impairs adipocyte identity in a cell-autonomous manner.

To further analyze the functional effects of *Lats1/2* deletion on AT in adult mice, we generated inducible adipocyte-specific *Lats1/2* double-knockout mice by crossing *Lats1*^*f/f*^*Lats2*^*f/f*^ with *Adipoq*^*Cre-ERT2*^ mice, named *Lats1*^*f/f*^*Lats2*^*f/f*^*Adipoq*^*Cre-ERT2*^ (L1L2-iAKO). We injected tamoxifen into L1L2-iAKO male mice to induce *Lats1/2* deletion at the age of eight weeks when adipocytes were well differentiated. Tamoxifen administration resulted ∼65% reduction of *Lats1* and *Lats2* expression in adipocyte rather than SVF from L1L2-iAKO scWAT (Supplementary Fig. 5f). Four weeks post tamoxifen administration, L1L2-iAKO mice exhibited impaired adipocyte identity with no alteration of body weight compared to L1L2-FF controls, as shown by the decreased expression of *Pparg*, *Fabp4*, *Adipoq*, and *Glut4* (Supplementary Fig. 5g, h). However, unlike what was observed in L1L2-AKO mice, the expression of fibrotic genes, including *Col1a1* and *Mmp14*, was nearly unaffected in L1L2-iAKO mice, albeit with a slight upregulation of *Fn1*, an early constituent of newly formed ECM (Supplementary Fig. 5i)^26^. We next sought to validate whether YAP and/or TAZ, the major LATS1/2 downstream effectors, mediates the fat-loss and/or fibrosis phenotype in adult mice. To target mature adipocytes, we developed an adiponectin promoter-derived (ADP) vector that drove gene expression specifically in three fat depots with the highest efficiency in scWAT (Supplementary Fig. 5j–l). Likewise, overexpression of fully active form of TAZ (4SA) or YAP (5SA), which contain gain-of-function mutations on the major phosphorylation sites^27^, resulted in evident fat loss but no fibrosis (Supplementary Fig. 5m–q). Thus, despite causing severe fat loss, Hippo pathway inactivation alone was not sufficient to activate fibrotic responses in AT of adult mice. These results reveal that AT fibrosis is not simply the consequence of fat loss and other signals are required for *Lats1/2* deficiency-initiated AT fibrosis.

### TGFβ stimulation coordinates with Hippo pathway inactivation to promote AT fibrosis

**Fig. 2:**
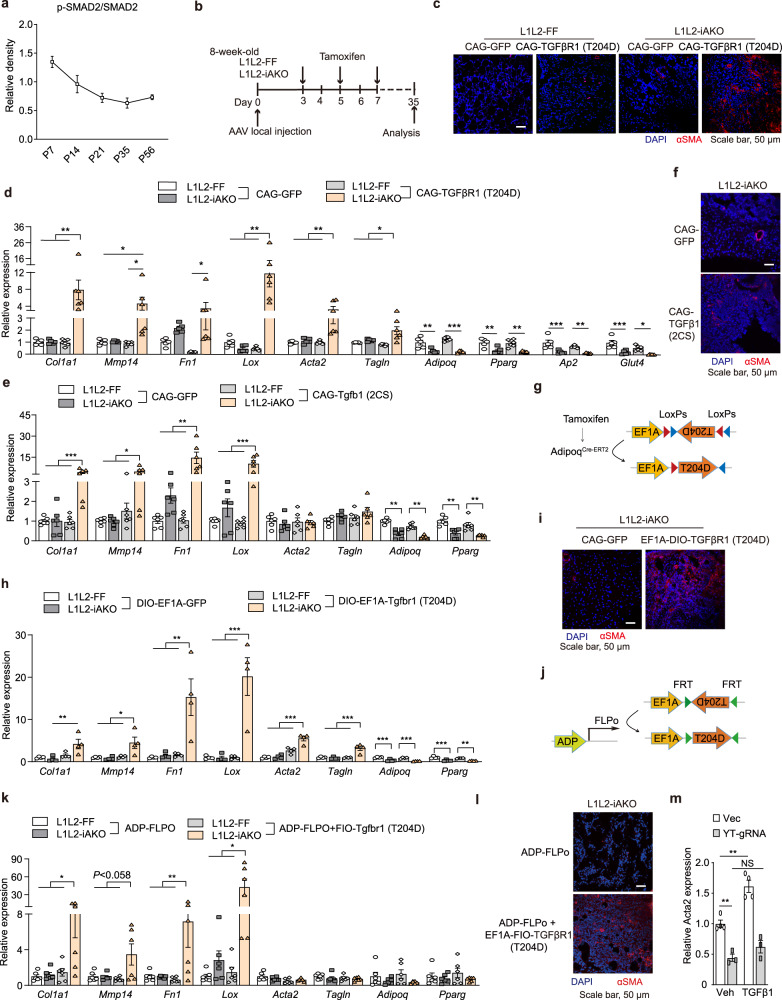
TGFβ stimulation is coupled with Hippo pathway inactivation to promote AT fibrosis. **a** Quantification of phosphorylation levels of SMAD2 in scWAT during growth (*n* = 4 mice). **b** Eight-week-old male L1L2-FF or *Lats1*^*f/f*^*Lats2*^*f/f*^*Adipoq*^*CreERT2*^ (L1L2-iAKO) mice were locally injected with AAV-CAG-GFP and AAV-CAG-TGFβR1 (T204D) respectively in scWAT. After 3 days, all mice were intraperitoneally (i.p.) administered 3 doses of tamoxifen every other day and then analyzed 4 weeks later. **c** Immunostaining of αSMA^+^ cells in the indicated mice. Independent experiments were performed twice with similar results. **d** mRNA expression of fibrotic and adipocyte markers in L1L2-FF or L1L2-iAKO scWAT transduced with AAV-CAG-GFP (*n* = 5 mice) or AAV-CAG-TGFβR1 (T204D) (*n* = 6 mice) followed by tamoxifen administration. **e**, **f** L1L2-FF or L1L2-iAKO mice were locally injected with AAV-CAG-TGFβ1 (2CS) in scWAT. **e** mRNA expression of fibrotic and adipocyte markers (*n* = 5 mice). **f** Immunostaining of αSMA^+^ cells. **g** AAV vectors for inducible expression of TGFβR1 (T204D) by Double-Floxed Inverted Open reading frame system. **h**, **i**, mRNA expression of fibrotic and adipocyte markers (**h**) (*n* = 4 mice) or immunostaining of αSMA^+^ cells (**i**) in L1L2-FF or L1L2-iAKO scWAT transduced with AAV-CAG-GFP or AAV-EF1A-DIO-TGFβR1. **j** Eight-week-old male L1L2-FF and L1L2-iAKO mice were subcutaneously co-injected with AAV-ADP-FLPo and AAV-EF1A-FIO-TGFβR1 (T204D) for 3 weeks, followed by i.p. injection of tamoxifen. **k**, **l** mRNA expression of fibrosis markers (**k**) (*n* = 6 mice) or immunostaining of αSMA^+^ cells (**l**) of mice in **j**. **m** mRNA expression of *Acta2* of *Cas9*^*Tg/+*^ SVF transduced with vector (*n* = 4 biologically independent cell cultures) or YT-gRNA (*n* = 3 biologically independent cell cultures) in the presence or absence of TGFβ1 for 36 h. Data are means ± SEM. Two-way analysis of variance (ANOVA) with Bonferroni’s multiple-comparisons test in **d**, **e**, **h**, **k**, **m**; *P < 0.05, **P < 0.01, ***P < 0.001; NS, not significant. Exact *P* values are provided in a Source data file.

We next sought to explore why Hippo pathway inactivation triggers both fat loss and AT fibrosis in L1L2-AKO but not in L1L2-iAKO mice. The phosphorylation levels of SMAD2 were decreased during mouse growth (Fig. 2a and Supplementary Fig. 6b) and the nuclear localization of p-SMAD2 was enriched at P7 and P14 (Supplementary Fig. 6a). The mRNA expression of *Tgfbr1* and *Tgfbr2* in scWAT was significantly higher in newborn mice (1-2 weeks old) than in adult mice (Supplementary Fig. 6c, d). These data indicated that the TGFβ pathway is active at early time points. CD9 serves as a co-receptor for TGFβ signaling in melanoma^28^. We found that *CD9* mRNA expression was down-regulated in scWAT during growth, and it was increased in L1L2-AKO pups but not in L1L2-iAKO adults (Supplementary Fig. 6e–g). Furthermore, TGFβ signaling was strongly activated in L1L2-AKO mice, as evidenced by a robust increase of *Tgfb* mRNA expression and phosphorylation of TGFβ’s downstream transcriptional factor SMAD2 (Fig. 1m and Supplementary Fig. 6h). By contrast, the expression of *Tgfb1/2/3* and *Tgfbr1/2* mRNA did not alter in L1L2-iAKO scWAT *(*Supplementary Fig. 6i*)*, suggesting that TGFβ signaling was unaffected in L1L2-iAKO adults. In addition, *Tgfb1/2/3* were not induced in *Lats1/2*-deficient adipocytes, suggesting that, unlike in liver, TGFβ signaling is not a downstream effector of YAP/TAZ activation in AT^29^ (Supplementary Fig. 6j). We further examined the involvement of the non-canonical TGFβ pathways mediated by MAP kinases (ERK, JNK1/2, p38), PI3K/AKT and Rho-ROCK-MLC2, and observed that only JNK1/2 were activated in L1L2-AKO scWAT (Supplementary Fig. 6k). However, JNK1/2 were also activated in L1L2-iAKO scWAT (Supplementary Fig. 6l), suggesting that the JNK pathway does not contribute to AT fibrosis. Deletion of *Lats1* and *Lats2* in L1L2-iAKO mice started at P2 did not alter body weight at 5 weeks old, but did induce AT fibrosis (Supplementary Fig. 6m–q). We thus speculated that weak TGFβ signaling might account for the lack of AT fibrosis in L1L2-iAKO adult mice.

To test this possibility, we first overexpressed TGFβRI (T204D), a constitutively active form of type I TGFβ receptor that activates TGFβ signaling in the absence of type II receptor^30^, followed by tamoxifen administration to induce *Lats1/2* deletion in scWAT (Fig. 2b and Supplementary Fig. 7a, b). Constitutive activation of TGFβRI could induce severe fibrotic responses, as shown by increased expression of fibrotic genes and increased numbers of αSMA^+^ myofibroblasts in L1L2-iAKO mice in comparison with L1L2-FF mice (Fig. 2c, d). Notably, overexpression of TGFβRI (T204D) in L1L2-FF mice did not influence adipocyte identity and fibrosis albeit elevating SMAD2 phosphorylation and nuclear localization of p-SMAD2 in scWAT (Fig. 2d and Supplementary Fig. 7c, h–k), indicating that activation of TGFβ signaling alone is not sufficient to induce AT fibrosis.

To confirm the importance of TGFβ signaling in Hippo pathway inactivation-elicited AT fibrosis, we overexpressed active mature TGFβ1 (2CS) in scWAT (Supplementary Fig. 7d, e), which was achieved by converting Cys223 and Cys225 to serine in TGFβ1 propeptides^31^, and observed an induction of fibrotic program in tamoxifen-administrated L1L2-iAKO adult mice compared to L1L2-FF controls (Fig. 2e, f). To address whether *Lats1/2*-deficient adipocytes responded to TGFβ signal, we performed inducible expression of TGFβRI (T204D) specifically in adipocytes using Cre-ERT2-mediated DIO system (Fig. 2g and Supplementary Fig. 7f). After overexpression of TGFβRI (T204D) in adipocytes, L1L2-iAKO mice showed elevated fibrotic responses compared to controls (Fig. 2h, i). Consistently, using FLPo-mediated FIO system to overexpress TGFβRI (T204D) specifically in adipocytes, we observed similar phenotypes in L1L2-iAKO mice (Fig. 2j–l and Supplementary Fig. 7g). In addition, TGFβ treatment upregulated *Acta2* expression in adipose precursor cells, while this effect was abrogated in YAP/TAZ-deficient precursor cells (Fig. 2m and Supplementary Fig. 7k). These findings suggest that Hippo pathway inactivation is coupled with TGFβ stimulation to synergistically promote AT fibrosis.

**Fig. 3:**
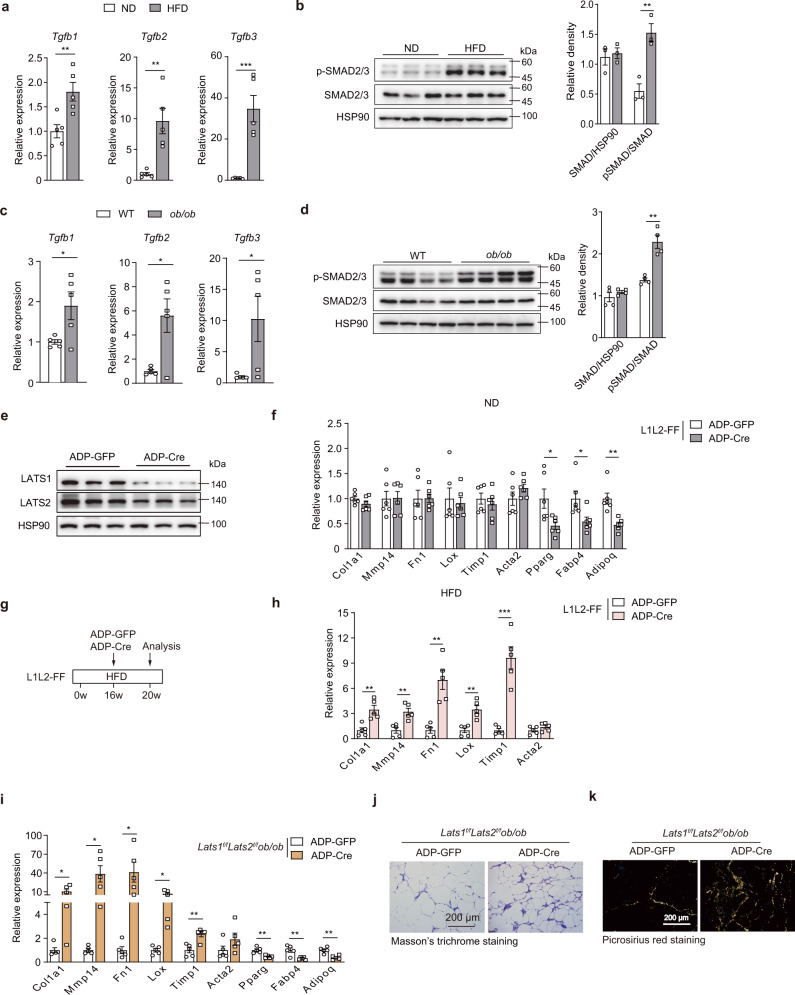
*Lats1/2* deficiency induces AT fibrosis in obese mice. **a** mRNA expression of *Tgfb1/2/3* in scWAT of male mice fed a ND or HFD for 18 weeks from 6 weeks old (*n* = 5 mice). **b** Immunoblot analysis of phosphorylation of SMAD2/3 (p-SMAD2/3) of mice fed a ND or HFD for 18 weeks. Right, quantification of protein and relative phosphorylation levels of SMAD2/3 (*n* = 3 mice). **c** mRNA expression of *Tgfb1/2/3* in scWAT of 12-week-old male WT or *ob/ob* mice (*n* = 5 mice). **d** Immunoblot analysis of protein expression of p-SMAD2/3 of mice in 12-week-old male WT or *ob/ob* mice. Right, quantification of protein and relative phosphorylation levels of SMAD2/3 (*n* = 4 mice). **e**, **f** Eight-week-old male *Lats1*^*f/f*^*Lats2*^*f/f*^ mice were injected with AAV-ADP-GFP or AAV-ADP-Cre in scWAT for 4 weeks. **e** Immunoblot analysis of LATS1 or LATS2 knockout efficiency in scWAT. **f** mRNA expression of fibrotic and adipocyte markers in scWAT (*n* = 6 mice). **g** Experimental outline: male L1L2-FF mice were fed a HFD for 16 weeks from 6 weeks old, followed by a local injection with AAV-ADP-GFP or AAV-ADP-Cre in scWAT, and all were analyzed after 4 weeks. **h** RT-qPCR analysis of fibrosis markers in scWAT of mice in **g** (*n* = 5 mice). **i** RT-qPCR analysis of fibrosis markers in scWAT of 12-week-old male *Lats1*^*f/f*^*Lats2*^*f/f*^*ob/ob* mice injected with AAV-ADP-GFP or AAV-ADP-Cre (*n* = 5 mice). **j**, **k** Representative scWAT sections with Masson’s trichrome staining (**j**) or Picrosirius red staining (**k**) of mice in **i**. Data are means ± SEM. Two-tailed unpaired Student’s *t* test; *P < 0.05, **P < 0.01, ***P < 0.001. Exact *P* values are provided in a Source data file.

TGFβ signaling was activated in obesity (Fig. 3a–d), consistent with what was previously reported^32^. We thus investigated whether *Lats1/2* deficiency could exacerbate fat tissue fibrosis in the context of obesity that provides endogenous TGFβ signal. To this end, we knocked out *Lats1/2* in scWAT of adult L1L2-FF mice using AAV-ADP-Cre (Fig. 3e). *Lats1/2* deletion impaired adipocyte identity but did not alter fibrotic gene expression in mice fed a normal diet (ND) (Fig. 3f), whereas *Lats1/2* deficiency induced fibrotic gene expression and altered ECM deposition when fed a HFD (Fig. 3g, h). Consistently, *Lats1/2* deficiency further increased ECM accumulation in the genetic obese mice (*Lats1*^*f/f*^*Lats2*^*f/f*^*ob/ob*) (Fig. 3i–k). Collectively, these findings support the critical role of two-signal regulatory circuit (Hippo pathway inactivation coupled with TGFβ stimulation) in the progression of AT fibrosis.

### *Lats1/2* deficiency elicits a CCL2/CCL7-macrophage feedforward loop to increase TGFβ expression

**Fig. 4:**
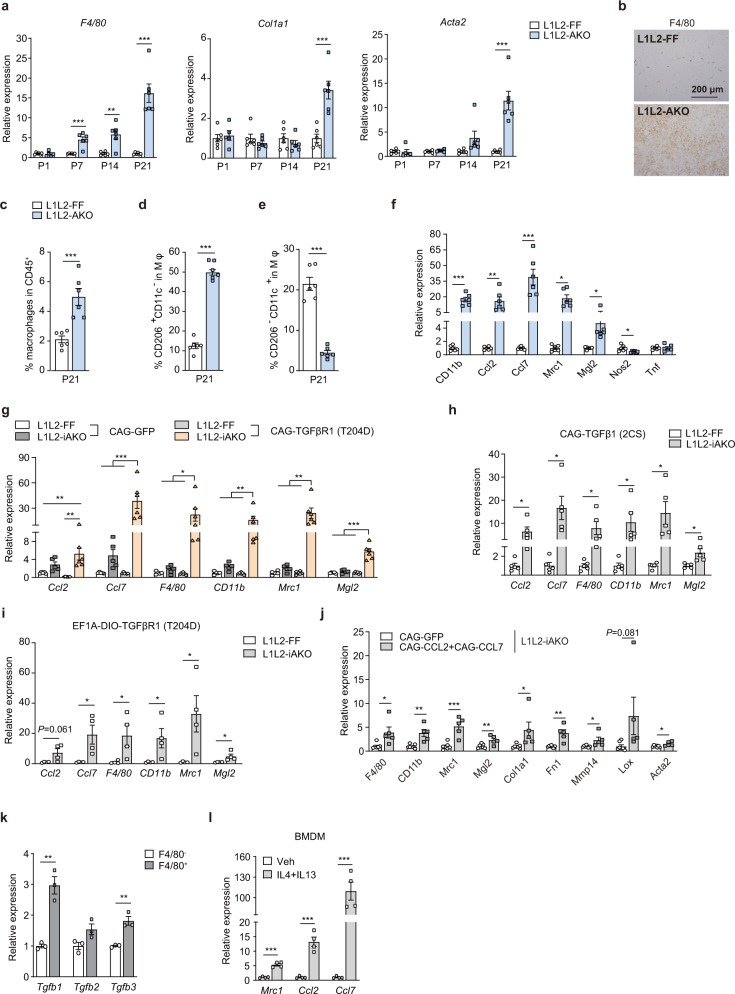
*Lats1/2* deficiency in adipocytes elicits a CCL2/CCL7-macrophage feedforward loop that increases TGFβ expression in macrophages. **a** Expression of *F4/80*, *Col1a1*, and *Acta2* of L1L2-FF (*n* = 6) and L1L2-AKO (*n* = 5 at P1, *n* = 6 at P7, P14 and P21) mice during growth. **b** Representative scWAT sections stained for F4/80 of 3-week-old male L1L2-FF or L1L2-AKO mice. Independent experiments were performed three times with similar results. **c** Quantification of F4/80^+^ cell percentages in scWAT SVF of 3-week-old male L1L2-FF or L1L2-AKO mice (*n* = 6). **d**, **e** Quantification of CD206^+^CD11c^−^ (**d**) and CD206^−^CD11c^+^ (**e**) cell percentages in scWAT SVF of 3-week-old male L1L2-FF or L1L2-AKO mice (*n* = 6). **f** mRNA expression of inflammatory markers in scWAT of 3-week-old male L1L2-FF or L1L2-AKO mice (*n* = 6). **g** mRNA expression of inflammatory genes in male L1L2-FF and L1L2-iAKO mice that were locally injected with AAV-CAG-GFP (*n* = 5) or AAV-CAG-TGFβR1 (T204D) (*n* = 6) in scWAT. **h** mRNA expression of inflammatory genes in male L1L2-FF or L1L2-iAKO mice that were locally injected with AAV-CAG-TGFβ1 (2CS) in scWAT (*n* = 5). **i** mRNA expression of inflammatory genes in male L1L2-FF or L1L2-iAKO mice that were locally injected with AAV-EF1A-DIO-TGFβR1 (T204D) in scWAT (*n* = 4). **j** Eight-week-old male L1L2-iAKO mice were subcutaneously injected with AAV-CAG-GFP (*n* = 7) or AAV-CAG-CCL2/CCL7 (*n* = 5) and analyzed for mRNA expression of inflammatory genes and fibrosis markers 4 weeks later. **k** F4/80^−^ and F4/80^+^ cells were magnetically sorted from pooled WT scWAT SVF and analyzed for mRNA expression of *Tgfb1/2/3* (*n* = 3). scWAT from three male mice were pooled as one sample. **l** BMDMs were treated with Veh, and IL4 plus IL13 for 24 h and analyzed for *Mrc1*, *Ccl2* and *Ccl7* mRNA expression (*n* = 4 biologically independent cell cultures). Data are means ± SEM. Two-tailed unpaired Student’s *t* test in **a**, **c**–**f**, **h**–**l**; two-way ANOVA with Bonferroni’s multiple-comparisons test in **g**; *P < 0.05, **P < 0.01, ***P < 0.001. Exact *P* values are provided in a Source data file.

We analyzed the expression of *F4/80*, *Col1a1* and *Acta2* at P7, P14 and P21, which represent different stages of AT fibrosis (Fig. 1k). Noticeably, the mRNA expression of *F4/80* from L1L2-AKO scWAT started to exceed that of L1L2 controls at P7, earlier than that for *Col1a1* and *Acta2* at P21 (Fig. 4a). These observations suggested a possibility that inflammatory leukocytes may play a critical role in adipocyte-specific *Lats1/2* deficiency-elicited fibrotic responses. Indeed, we visualized a dramatic accumulation of macrophages in scWAT of L1L2-AKO mice by immunohistochemistry staining of F4/80 (Fig. 4b). Flow cytometry analysis showed a marked enrichment of macrophages in L1L2-AKO scWAT compared to those in L1L2-FF controls (Fig. 4c and Supplementary Fig. 8a). Macrophages are experimentally divided into classically activated (M1) and alternatively activated (M2) macrophages, based on their polarization state. Notably, the proportion of M2 (CD206^+^CD11c^−^) macrophages was robustly increased in L1L2-AKO scWAT (Fig. 4d), whereas that of M1 (CD11c^+^CD206^−^) macrophages was dramatically decreased (Fig. 4e). We also observed a robustly increased mRNA expression of M2 markers (*Mrc1* and *Mgl2*) and a decreased mRNA expression of M1 marker (*Nos2*) (Fig. 4f). These results suggest that M2 macrophages are involved in the regulation of fibrotic responses upon *Lats1/2* deficiency. In support of this point, a large number of macrophages, particularly M2 macrophages, infiltrated into fibrotic L1L2-iAKO AT upon TGFβ activation (Fig. 4g–i and Supplementary Fig. 8b), whereas no macrophage recruitment occurred in non-fibrotic AT (Supplementary Fig. 8c).

We next investigated how macrophages infiltrate into scWAT upon *Lats1/2* deletion. CCL2 and CCL7 are essential chemokines that recruit monocytes^33^. *Ccl2* expression was up-regulated in L1L2-iAKO scWAT and in L1L2-AKO adipocytes (Supplementary Fig. 8d, e), which was congruent with a previous report showing that YAP transcriptionally enhanced *Ccl2* expression through binding to its promoter^34^. We simultaneously overexpressed CCL2 and CCL7 in scWAT of L1L2-iAKO mice (Supplementary Fig. 8f), and observed a significant upregulation of marker gene expression of monocytes and macrophages (particularly M2 macrophages) compared to the controls, accompanied by an increase of fibrotic gene expression and ECM accumulation (Fig. 4j). These data suggest that *Lats1/2* deletion-elicited CCL2/CCL7 secretion recruits monocytes to differentiate into macrophages, particularly M2 macrophages. Unexpectedly, Hippo-inactivation induced CCL2/CCL7 expression in adipocytes was insufficient to recruit macrophages and promote fibrosis in adult L1L2-iAKO mice (Supplementary Figs. 8d, 5i), indicating other cell compartments might produce CCL2/CCL7. Compared to F4/80^−^ stromal fractions, F4/80^+^ macrophages expressed more *Ccl2* and *Ccl7* in L1L2-AKO mice (Supplementary Fig. 8g, h). In addition, F4/80^+^ macrophages expressed more *Tgfb* than F4/80^−^ cells of scWAT (Fig. 4k), indicating macrophages contributed to TGFβ production. M2 polarization of BMDM by IL4 and IL13 co-treatment elevated its *Ccl2* and *Ccl7* expression (Fig. 4l). Together, these data imply that macrophages could express CCL2/CCL7 to further amplify macrophage recruitment, i.e, forming a “CCL2/CCL7-macrophage” feedforward and thus produce more TGFβ to enhance fibrotic responses. However, due to reduced type 2 immunity and TGFβ signaling in adult scWAT (Fig. 2a)^35^, the feedforward loop cannot work efficiently, which explains why *Lats1/2* knockout in adult mice leads to fat loss alone but no fibrosis.

### Adipocytes dedifferentiate into DPP4^+^ progenitors and convert to DPP4^−^ myofibroblasts upon *Lats1/2* loss

**Fig. 5:**
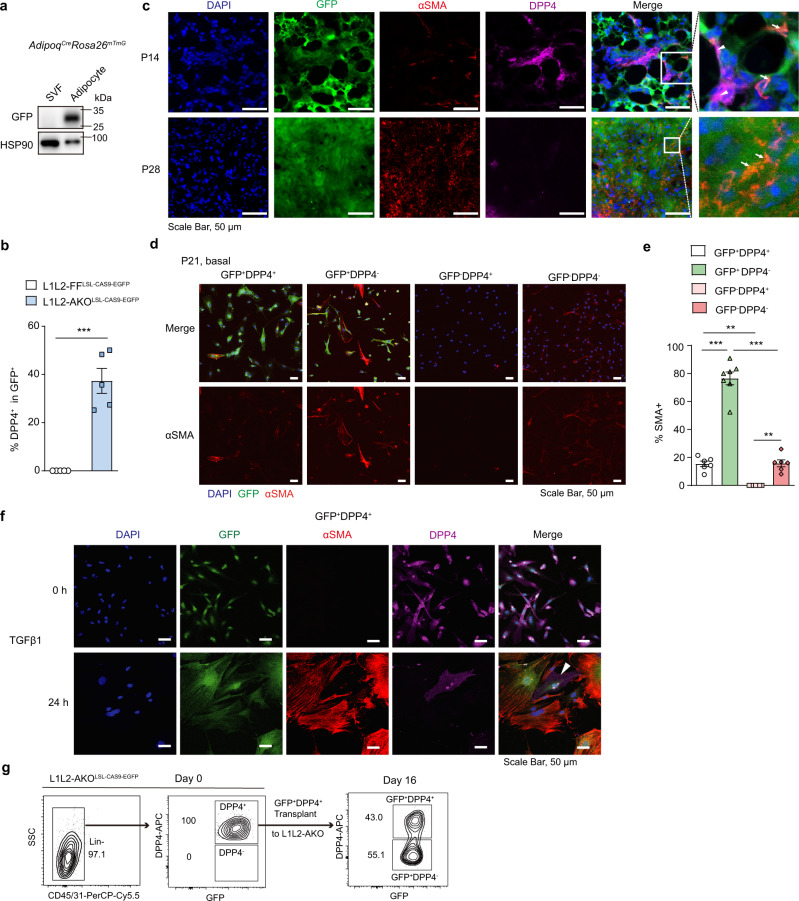
*Lats1/2* deficiency promotes cell fate conversion from adipocytes to myofibroblasts. **a** Immunoblot analysis of protein expression of GFP in SVF and adipocyte from 8-week-old *Adipoq*^*Cre*^*Rosa26*^*mTmG*^ scWAT. Independent experiments were performed twice with similar results. **b** Flow cytometry analysis of percentages of live CD45^−^CD31^−^GFP^+^DPP4^+^ cells in scWAT SVF of P21 L1L2-FF^LSL-CAS9-EGFP^ or L1L2-AKO^LSL-CAS9-EGFP^ reporter mice (*n* = 5). **c** Representative sections of scWAT of P14 or P28 L1L2-AKO^LSL-CAS9-EGFP^ mice stained for αSMA (red), DPP4 (purple), nucleus (DAPI, blue) and GFP (green). Arrowheads indicate examples of GFP^+^DPP4^+^ cells. Arrows point to GFP^+^αSMA^+^ cells. Independent experiments were performed three times with similar results. **d** Sorted cell subsets from P21 L1L2-AKO^mTmG^ scWAT SVF were plated and stained for αSMA (AF647, red) and nucleus (DAPI, blue). **e** Quantification of percentages of GFP+DPP4+ (*n* = 6), GFP+DPP4- (*n* = 7), GFP-DPP4+ (*n* = 7), GFP-DPP4- (*n* = 7) cells. Dots represent cell percentages of random visual fields from P21 L1L2-AKOmTmG mice. Independent experiments were performed twice with similar results. **f** Sorted GFP^+^DPP4^+^ cells from P21 L1L2-AKO^LSL-CAS9-EGFP^ scWAT SVF were treated with TGF-β1 (10 ng/ml) for 0 h or 24 h. Cells were stained with αSMA (red), DPP4 (purple), nucleus (DAPI, blue) and GFP (green). An arrowhead points to the GFP^+^αSMA^−^DPP4^+^ cell. Independent experiments were performed twice with similar results. **g** Sorted GFP^+^DPP4^+^ cells from L1L2-AKO^LSL-CAS9-EGFP^ reporter scWAT SVF were analyzed for DPP4 expression in GFP^+^ cells before or after transplantation into scWAT of 8-day-old L1L2-AKO recipient mice. One representative transplant from *n* = 3 biological replicates. Data are means ± SEM. One-way ANOVA with Bonferroni’s multiple-comparisons test in **e**; two-tailed unpaired Student’s *t* test in **b**. *P < 0.05, **P < 0.01, ***P < 0.001. Exact *P* values are provided in a Source data file.

Myofibroblasts, marked by αSMA expression, are important effector cells that make ECM molecules to aggravate fibrosis, and they are differentiated from fibroblast-type cells by TGFβ stimulation^36^. Fibrosis and adipogenesis have been shown to be mutually exclusive during adipose precursor commitment^12,37–39^. Given the adipocyte identity loss and robust fibrosis upon *Lats1/2* deletion, we hypothesized that *Lats1/2*-deficient adipocytes convert into myofibroblasts to aggravate fibrosis. To test this idea, we crossed *Lats1*^*f/+*^*Lats2*^*f/f*^*Adipoq*^*Cre*^ with *Rosa26*^*mTmG*^ reporter mice to generate *Lats1*^*f/f*^*Lats2*^*f/f*^*Adipoq*^*Cre*^*Rosa26*^*mTmG*^ mice (L1L2-AKO^mTmG^), which allowed us to trace *Lats1/2*-deficient adipocytes as they are GFP^+^ (Supplementary Fig. 9a). *Adipoq*^*Cre*^*Rosa26*^*mTmG*^ model was shown to specifically target mature adipocyte but not adipose precursors in postnatal scWAT (Fig. 5a)^40^. As expected, a high proportion of cells in scWAT exhibited as GFP^+^ cells which comprised a large percentage of αSMA^+^ cells, indicating that many *Lats1/2*-deficient adipocytes resided in situ were converted into αSMA^+^ cells (Supplementary Fig. 9a). GFP^+^ cells gained proliferative capacity, as shown by the considerable presence of GFP^+^Ki-67^+^ cells in L1L2-AKO mice and the high cell density appearance of fibrotic AT (Supplementary Fig. 9b and Fig. 1k). In sharp contrast, no αSMA^+^ myofibroblast was observed in the scWAT of L1L2-iAKO^mTmG^ mice in which fibrosis and adipocyte loss were uncoupled (Supplementary Fig. 9c), indicating that a direct transdifferentiation from adipocyte to myofibroblast was unlikely. Notably, adipocyte-derived myofibroblasts appeared only upon TGFβ activation (Supplementary Fig. 9d).

Previous studies have established that DPP4 serves as an adipose progenitor marker in scWAT^41^ and DPP4^−^ preadipocytes are enriched with fibrotic genes in obese mice^14^. In addition, DPP4^+^ cells exhibited less αSMA expression compared to DPP4^−^ in WT scWAT (Supplementary Fig. 9e, f), implying that DPP4 may serve as a cell surface marker to distinguish the fibrotic potential of adipose progenitors. Based on these observations, we hypothesized that adipocytes dedifferentiate into DPP4^+^ progenitor cells followed by more committed DPP4^−^ cells. Flow cytometry analysis of stromal cells shows that 37% of GFP^+^ cells were DPP4^+^ cells within the scWAT of L1L2-AKO mice (Fig. 5b). Notably, GFP^+^ cells colocalized with DPP4^+^ cells as early as 2 weeks old when adipocytes started to lose their identity in L1L2-AKO^LSL-CAS9-EGFP^ mice (Fig. 5c and Supplementary Fig. 9g). By contrast, the proportion of GFP^+^DPP4^+^ cells reduced while that of GFP^+^DPP4^−^ cells increased at 4 weeks old as AT fibrosis progressed (Fig. 5c), and the proliferation capacity of GFP^+^DPP4^+^ was greater than that of GFP^+^DPP4^−^ cells (Supplementary Fig. 9h, i). Consistently, deletion of *Lats1/2* in differentiated adipocytes induced *Dpp4* expression in vitro (Supplementary Fig. 9j), indicating that *Lats1/2*-deficient adipocytes could convert to DPP4^+^ cells in a cell autonomous manner.

To investigate the cell fate conversion, we first isolated and plated four cell populations from P21 L1L2-AKO^mTmG^ scWAT, which were GFP^+^DPP4^+^, GFP^+^DPP4^−^, GFP^−^DPP4^+^ and GFP^−^DPP4^−^ cells, and examined αSMA expression to determine which population has greater fibrotic potential. Interestingly, the percentages of DPP4^−^ cells expressing αSMA were much higher than those of DPP4^+^ cells (Fig. 5d, e). Dedifferentiated GFP^+^ cells became more fibrotic, as revealed by higher percentages of GFP^+^ cells expressing αSMA than those of respective GFP^−^ cells (Fig. 5d, e). These results suggest that myofibroblasts majorly derive from DPP4^−^ progenitors, particularly GFP^+^DPP4^−^ ones. We next sought to explore whether DPP4^+^ progenitors could convert to DPP4^−^ cells, and found that DPP4 did not colocalize with αSMA in GFP^+^DPP4^+^ cells isolated from P21 L1L2-AKO^LSL-CAS9-EGFP^ scWAT, whereas a subset of DPP4^−^ cells expressed αSMA in quiescent condition (Supplementary Fig. 9k). Upon TGFβ stimulation, nearly all GFP^+^DPP4^+^ cells were converted into GFP^+^DPP4^−^ cells, which expressed high level of αSMA (Fig. 5f). To validate this cell fate conversion in vivo, we transplanted GFP^+^DPP4^+^ cells into scWAT of L1L2-AKO recipient mice. Nearly half of the transplanted GFP^+^DPP4^+^ cells lost DPP4 expression by day 16 (Fig. 5g and Supplementary Fig. 9l). Collectively, these findings suggest that DPP4^+^ cells can convert to DPP4^−^ myofibroblasts.

### The YAP/TAZ–TEADs axis cooperates with SMAD2 to enhance fibrotic responses

**Fig. 6:**
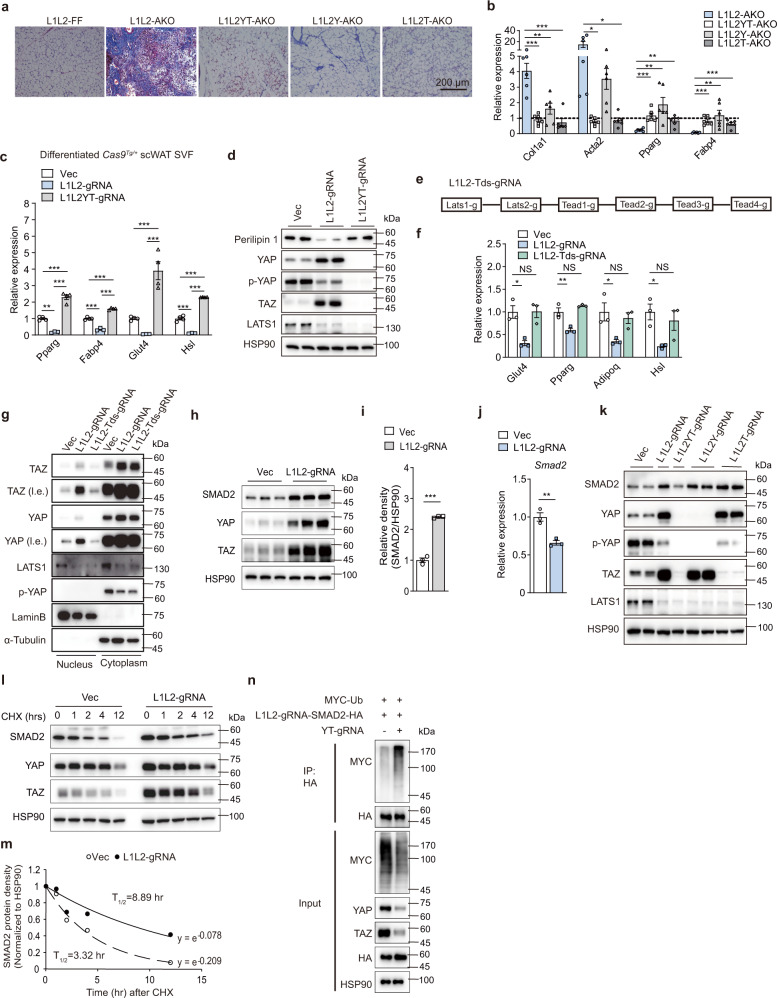
The YAP/TAZ–TEADs axis cooperates with SMAD2 to regulate fibrotic responses. **a** Representative scWAT sections with Masson’s trichrome staining of indicated mouse strains at 5 weeks old. Independent experiments were performed three times with similar results. **b** mRNA expression of fibrosis and adipocyte markers (*n* = 6 mice). Data are shown as fold change in L1L2-AKO, L1L2YT-AKO, L1L2Y-AKO, and L1L2T-AKO compared to corresponding WT littermates. The column of L1L2-AKO had also been shown in Fig. 1m. **c**–**n** Differentiated adipocytes from *Cas9*^*Tg/+*^ scWAT was transduced with the indicated gRNAs on day 2 post differentiation, and then analyzed 5 days later. **c** mRNA expression of adipocyte markers in differentiated adipocytes transduced with Vec (*n* = 4 biologically independent cell cultures), *Lats1/2* gRNA (L1L2-gRNA) (*n* = 3 biologically independent cell cultures) and LATS1/2-YAP/TAZ gRNA (L1L2YT-gRNA) (*n* = 4 biologically independent cell cultures). **d** Immunoblot analysis of protein expression in cells in **c**. **e** Schematic diagram of the *Lats1/2*-*Tead1/2/3/4* Tandem gRNA construct. **f** mRNA expression of adipocyte marker in differentiated adipocytes transduced with Vec, L1L2-gRNA, or *Lats1/2*-*Tead1/2/3/4* gRNA (L1L2-Tds-gRNA) (*n* = 3 biologically independent cell cultures). **g** YAP and TAZ protein level in the nuclear/cytoplasmic fractionation of differentiated adipocytes transduced with Vec, L1L2-gRNA and L1L2-Tds-gRNA. **h** Immunoblot analysis of protein expression of SMAD2, YAP and TAZ in differentiated adipocytes transduced with Vec and L1L2-gRNA. **i** Quantification of relative density of SMAD2 normalized to HSP90 (*n* = 3 biologically independent cell cultures). **j** mRNA expression of *Smad2* of cells in **i** (*n* = 3 biologically independent cell cultures). **k** Immunoblot analysis of SMAD2 protein expression in differentiated adipocytes (from SVF of *Cas9*^*Tg/+*^ scWAT) transduced with the indicated gRNAs. **l** Immunoblot analysis of SMAD2 protein expression in a cycloheximide chase experiment. **m**, Regression analysis of SMAD2 protein stability in **l**. **n** Differentiated adipocytes were transduced with MYC-Ub, L1L2-gRNA-SMAD2-HA and YT-gRNA. Immunoprecipitation (IP) assay showing an increased ubiquitination of SMAD2 in *Yap*/*Taz*-deficient cells. Data are means ± SEM. One-way ANOVA with Bonferroni’s multiple-comparisons test in **c**, **f**; two-tailed unpaired Student’s *t* test in **b**, **i**, **j**; *P < 0.05, **P < 0.01, ***P < 0.001; NS, not significant. Exact *P* values are provided in a Source data file.

To determine whether YAP/TAZ mediate the *Lats1/2* inactivation-elicited fibrotic phenotype, we crossed *Lats1*^*f/f*^*Lats2*^*f/f*^*Yap*^*f/f*^*Taz*^*f/f*^ (L1L2YT-FF) with *Adipoq*^*Cre*^ mice to simultaneously delete *Lats1*, *Lats2*, *Yap* and/or *Taz* in adipocytes (L1L2Y/T-AKO). The fibrotic phenotype elicited in L1L2-AKO was completely rescued in L1L2YT-AKO and L1L2T-AKO AT or largely rescued in L1L2Y-AKO AT, exhibiting undetectable or mild collagen deposition and less fibrotic gene expression (Fig. 6a, b). Deprived adipocyte identity by *Lats1/2* loss was fully rescued upon YAP/TAZ deletion (Fig. 6c, d and Supplementary Fig. 10a, b). Likewise, YAP/TAZ governed adipose precursor cell fate towards adipocyte or myofibroblast (Supplementary Fig. 10c–g). TEADs are major transcription factors mediating the transactivation function of YAP/TAZ. To examine whether TEADs mediated the adipocyte identity-loss phenotype, we employed a tandem guide RNA-based strategy to simultaneously knockout *Tead1-4* and *Lats1/2* in differentiated SVF cells from *Cas9*^*Tg/+*^ scWAT and confirmed the knockout efficiency by measuring mRNA expression of *Teads*, *Lats1/2* and their downstream targets (Supplementary Fig. 10h, i). *Lats1/2* deletion in vitro impaired adipocyte identity, which was fully rescued by TEADs knockout (Fig. 6e, f). Notably, YAP/TAZ accumulated in the nucleus upon *Lats1/2* deletion, whereas additional TEADs knockout abolished YAP/TAZ nuclear localization and transactivation function (Fig. 6g). Together, these results indicate that the impaired adipocyte identity by LATS1/2 inactivation was largely dependent on the activation of YAP/TAZ–TEADs axis, which was a prerequisite for the fibrotic responses.

SMADs are major effectors in response to TGFβ, and have been reported to regulate adiposity and energy homeostasis^15,32^. Loss of *Lats1/2* markedly increased the protein expression of SMAD2 as well as YAP and TAZ in adipocytes, while the mRNA expression of SMAD2 was decreased (Fig. 6h–j and Supplementary Fig. 10j). Similar changes were also observed in precursor cells from *Cas9*^*Tg/+*^ SVF or adipocytes differentiated from L1L2-FF and L1L2-AKO SVF (Supplementary Fig. 10k–p). Hippo inactivation-elicited increase of SMAD2 protein was dependent on the induction of YAP/TAZ expression (Fig. 6K, lane1–4; Supplementary Fig. 10q, r). In addition, YAP or TAZ induction alone was sufficient to increase the protein expression of SMAD2 (Fig. 6k, lane1, 2, and 5–8). Thus, YAP/TAZ may influence the stability of SMAD2 protein in a posttranslational manner. To test this hypothesis, we performed a cycloheximide chase experiment and found that SMAD2 protein had a longer half-life in *Lats1/2*-deficient adipocytes (Fig. 6l, m). To investigate how YAP/TAZ regulate SMAD2 stability, we examined whether YAP/TAZ affect SMAD2’s degradation by the ubiquitin–proteasome system (UPS). *Lats1/2* deletion decreased SMAD2 ubiquitination compared to the vector control (Supplementary Fig. 10s). Compared to *Lats1/2* knockout, additional *Yap/Taz* knockout dramatically increased ubiquitin modification of SMAD2 (Fig. 6n). Together, these findings indicate that the YAP/TAZ–TEADs axis cooperates with TGFβ stimulation to promote AT fibrosis, via an inhibitory regulation of the UPS to stabilize SMAD2.

### Targeting the YAP/TAZ–TEADs axis for treating AT fibrosis

**Fig. 7:**
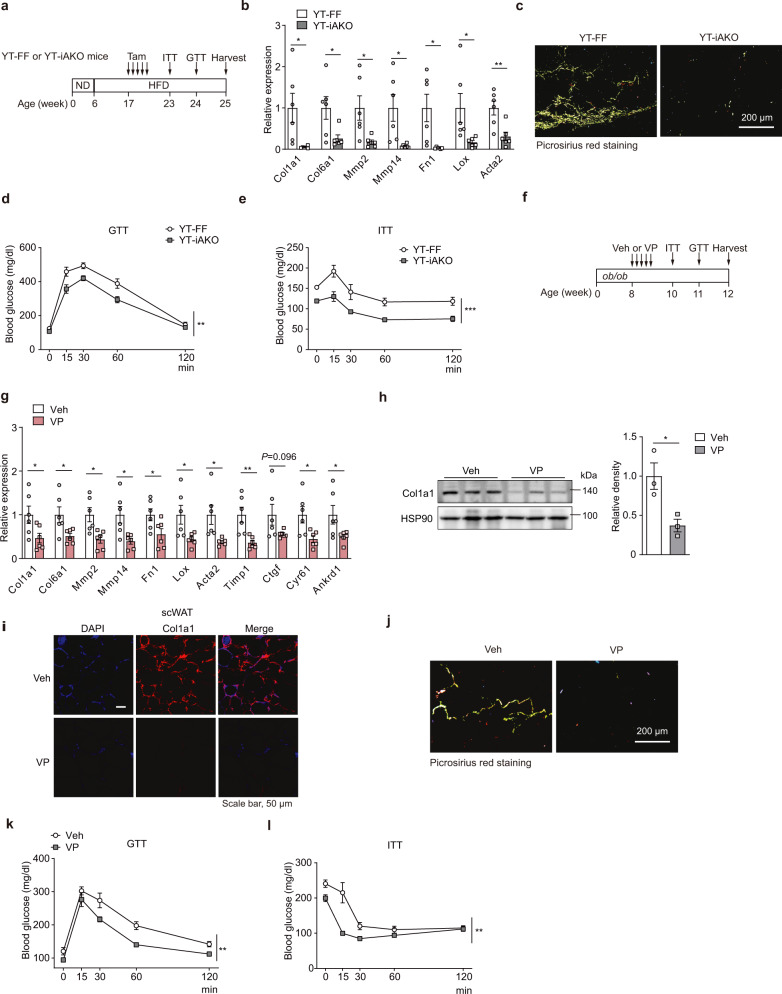
Targeting YAP/TAZ to relieve AT fibrosis in obese mice. **a**–**e** *Yap*^*f/f*^*Taz*^*f/f*^*Adipoq*^*CreERT2*^ (YT-iAKO) or YT-FF mice were fed a HFD for 11 weeks followed by i.p. injection of 5 doses of tamoxifen (50 mg/kg) every day (*n* = 6). **a** Experimental scheme. **b** mRNA expression of fibrosis markers in scWAT from YT-FF or YT-iAKO mice (*n* = 6). **c** Representative scWAT sections with Picrosirius red staining of YT-FF or YT-iAKO mice. **d**, **e** Glucose tolerance test (GTT) (**d**) or insulin tolerance test (ITT) (**e**) of YT-FF (*n* = 6) or YT-iAKO (*n* = 7) mice. **f**–**l** Eight-week-old *ob/ob* mice were i.p. injected with Veh or verteporfin (VP) (25 mg/kg) for 5 doses every other day. **f** Experimental scheme. **g** mRNA expression of fibrosis markers and YAP target genes in scWAT of *ob/ob* mice (*n* = 6). **h** Immunoblot analysis (left) and quantification (right) of Col1a1 (*n* = 3 mice). **i** Representative sections of scWAT stained for Col1a1 (red) and nucleus (DAPI, blue). **j** Representative scWAT sections with Picrosirius red staining of *ob/ob* mice. **k** GTT (*n* = 6 mice). **l** ITT (*n* = 6 mice). Data are means ± SEM. Two-tailed unpaired Student’s *t* test in **b**, **g**, **h**; two-way ANOVA with Bonferroni’s multiple-comparisons test in **d**, **e**, **k**, **l**; *P < 0.05, **P < 0.01, ***P < 0.001. Exact *P* values are provided in a Source data file.

To test the clinical implication of the YAP/TAZ–TEADs axis in AT fibrosis, we first crossed *Yap*^*f/f*^*Taz*^*f/f*^ (YT-FF) with *Adipoq*^*Cre-ERT2*^ mice to specifically knockout *Yap* and *Taz* in adipocytes (YT-iAKO) and fed a HFD to establish fibrosis before the inducible knockout of *Yap/Taz* (Fig. 7a). *Yap/Taz* inducible deletion in adipocytes drastically reduced fibrotic gene expression, ECM accumulation in scWAT and vWAT, but did not alter body weight (Fig. 7b, c and Supplementary Fig. 11a–d). Importantly, the attenuated AT fibrosis contributed to improved glucose intolerance and insulin resistance imposed by HFD-induced obesity (Fig. 7d, e), indicating that the deletion of YAP/TAZ could reduce AT fibrosis and associated metabolic dysfunction. Verteporfin has been identified to interrupt the YAP/TAZ-TEADs interaction and consequently block the transcriptional activation^42^. Thus, we used verteporfin to inactivate YAP/TAZ–TEADs axis. Administration with verteporfin resulted in a reduction of fibrotic gene expression and ECM accumulation and an improvement of metabolic homeostasis both in scWAT and vWAT of genetic *ob/ob* and HFD-induced obese mice (Fig. 7f–l and Supplementary Fig. 11e–j). On the other hand, we overexpressed the active TAZ derivative, TAZ(4SA), whose expression is controlled by adiponectin promoter and restricted in adipocytes. As expected, TAZ(4SA) overexpression exacerbated obesity-induced AT fibrosis, which is in contrast with a marked amelioration of fibrosis in *Yap/Taz*-iAKO obese mice (Supplementary Fig. 11k–q). Together, these findings suggest that targeting the YAP/TAZ–TEADs axis may effectively treat obesity-induced AT fibrosis and consequently improve metabolic function.

## Discussion

**Fig. 8:**
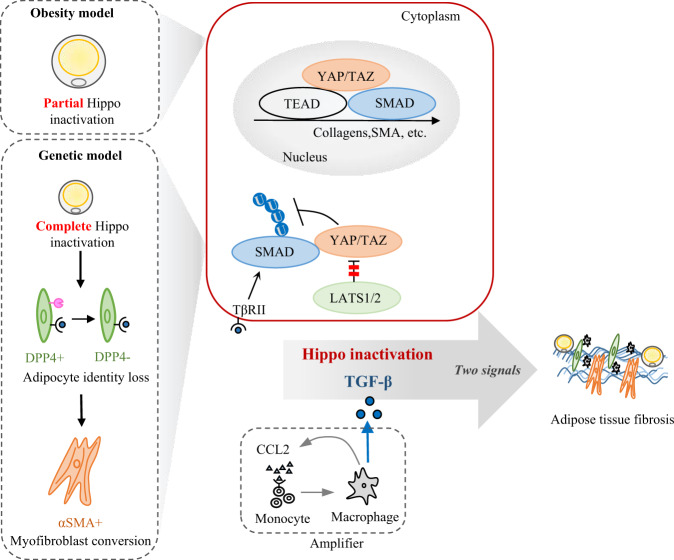
Hippo pathway and TGFβ signaling synergistically orchestrate adipocyte plasticity and AT fibrosis. In genetic model, *Lats1/2*-knockout adipocytes can dedifferentiate into DPP4^+^ progenitors and convert to DPP4^−^ myofibroblasts. Loss of *Lats1/2* activates YAP/TAZ that in turn enhance SMAD2 stability through inhibiting its ubiquitination. In the presence of CCL2 and CCL7, monocyte-derived macrophages are recruited to scWAT to produce TGFβ, which activates SMAD2 in dedifferentiated adipocytes to form a complex with YAP/TAZ. The complex translocates into the nucleus to induce transcription of inflammatory and fibrotic genes. In obesity model, the Hippo pathway is inhibited while TGFβ signaling is activated, which impairs adipocyte identity and promotes AT fibrosis. Moreover, CCL2/7 and macrophage form a feedforward loop to amplify inflammatory responses and accelerate AT fibrosis. Together, Hippo pathway inactivation is coupled with TGFβ stimulation to promote AT fibrosis.

Adipocyte plasticity and AT fibrosis are intertwined and play key roles in AT remodeling. In this study, we uncovered that the Hippo pathway and TGFβ signaling are coordinated in a “two-signal” manner to control adipocyte plasticity and AT fibrosis, which is modulated by fibroblast–immune cell interactions (Fig. 8). We found that adipocyte-specific *Lats1/2* deletion induces an adipocyte-to-myofibroblast transition to increase ECM accumulation. Specifically, *Lats1/2*-knockout adipocyte can de-differentiate into DPP4^+^ cells, and then convert to DPP4^−^ myofibroblast. On the other hand, Hippo pathway inhibition during obesity leads to a functional shift of adipocytes from energy storage to ECM remodeling. Macrophages produce TGFβ to promote AT fibrosis. Furthermore, CCL2/CCL7 and macrophage form a feedforward loop that acts as an amplifier to increase TGFβ expression and accelerate AT fibrosis (Fig. 8).

Our findings reveal that the Hippo pathway play an essential role in the maintenance of adipocyte identity and function, like PPARγ, CEBPα, PGC1α, PDGFRα and ZFP423^43^. Adiponectin-Cre expression has been shown in a subset of adipocyte precursors in fetal scWAT^44,45^, but restricted to mature adipocytes postnatally^40^. Notably, besides preadipocytes, we demonstrate that Hippo pathway can dramatically remodel the cell fate of terminally differentiated adipocytes, reminiscent of its essential role in the maintenance of liver cell fate^46^. PPARγ is considered as a master regulator of adipogenesis, which activates transcription of genes involved in the development of adipocytes, such as those encoding AP2 and adiponectin^47–51^. Moreover, TAZ has been reported to impair mature adipocyte function through binding to PPARγ and repressing its activity in vivo^52^. In our study, lipid-laden adipocytes existed in P14 L1L2-AKO scWAT (Fig. 1k), suggesting that preadipocyte can undergo normal adipogenesis process at least before P14. Thus, adipocyte loss upon *Lats1/2* deletion was attributed to dedifferentiation rather than impaired adipogenesis by the upregulated YAP/TAZ that has been shown to block adipogenic differentiation in vitro^21,22^. This is similar with the fat-loss phenotype of PPARγ adipocyte-specific knockout mice^47^. Together, these findings suggest that TAZ inhibits PPARγ activity to impair expression of genes that maintain adipocyte identity, and thus leads to adipocyte dedifferentiation.

Fibroblast heterogeneity exists across tissues^53^, raising an important question which fibroblast subsets contribute to AT fibrosis. It is appreciated that adipocytes, fibroblasts and inflammatory cells all play roles in AT fibrosis^10–13,54^. Interestingly, single-nucleus RNA-seq studies have shown that stressed lipid-scavenging adipocytes, a subpopulations of hypertrophic adipocytes, tend to impair their fat cell identity during the development of obesity, accompanied by an increased expression of genes related to immune response and collagen deposition^14^. We observed that *Lats1/2* deletion in adipocytes led to a complete fat loss, which provide an opportunity to better understand the role of adipocyte identity loss in the development of AT fibrosis. Indeed, we identified a subset of αSMA^+^ myofibroblasts that were originated from *Lats1/2*-deficient mature adipocytes. Furthermore, we showed that *Lats1/2* deletion promoted the conversion of DPP4^+^ progenitors to DPP4^−^ myofibroblasts. Thus, the Hippo pathway acts as a molecular switch in the cell fate commitment of DPP4^+^ progenitors toward either adipocytes or myofibroblasts, adding to the notion that DPP4^+^ progenitors exhibit multi-lineage differentiation potential^41^. However, we cannot exclude a possibility that adipocytes can directly dedifferentiate into DPP4^−^ cell and then convert to myofibroblasts.

Interestingly, unlike in newborn mice, adipocyte-specific *Lats1/2* knockout or *Yap/Taz* overexpression alone was not sufficient to induce fibrotic responses in adult mice, albeit with a massive fat loss. It might be attributed to a reduction of type 2 immunity and TGFβ signaling in AT during growth. On the other hand, TGFβ activation alone was not sufficient to induce fat loss, inflammation and AT fibrosis. However, TGFβ stimulation on dedifferentiated adipocytes of adult L1L2-iAKO mice could elicit AT fibrosis, recapitulating the phenotypes of newborn mice. These findings demonstrate that Hippo pathway links adipocyte plasticity to AT fibrosis in concert with TGFβ signaling (Fig. 8).

We further revealed that YAP/TAZ cooperate with SMADs to drive AT fibrosis, echoing with the inhibition effects of SMADs on adipogenesis^32^. Our results demonstrate that YAP/TAZ promote SMAD2 stability in a post-translational manner, thereby enhancing its sensitivity and endurance geared toward TGFβ stimulation. It has been well established that macrophages cooperate with fibroblasts to maintain tissue homeostasis, and their interaction is regulated by TGFβ in the development of fibrosis^55^. We found that macrophage recruitment preceded AT fibrosis, and macrophages produced TGFβ to promote AT fibrosis. Accordingly, CCL2 and CCL7 regulate macrophage infiltration to induce fibrotic responses in L1L2-iAKO mice. Our findings therefore uncover a “CCL2/7-macrophage” feedforward loop that accelerates fibrotic responses.

We have provided a proof-of-concept for the therapeutic implications of the YAP/TAZ–TEADs axis in AT fibrosis. Through inducible knockout of YAP/TAZ in adipocytes, mice became resistant to obesity-induced fibrosis and metabolic dysfunction. Furthermore, using verteporfin as a chemical genetic tool to disrupt the YAP/TAZ–TEADs axis after the onset of AT tissue fibrosis, we found that the obesity-induced fibrosis was largely ameliorated by the treatment. Verteporfin is an FDA-approved compound originally for the treatment of choroidal neovascularization in photodynamic therapy^56^. It has been reported to exert broad anti-fibrotic effects in different contexts, like wounded skin regeneration, hepatic stellate cell activation, renal fibrosis and myocardial fibrosis^57–60^. Thus, targeting the YAP/TAZ–TEADs axis holds great potential to treat obesity-induced AT fibrosis.

In this study, we employed a knockout mouse model to probe the cellular and molecular mechanisms underlying the initiation and development of adipose fibrosis upon pathological condition like severe obesity. However, the *Lats1/2* deletion causes severe lipodystrophy, which has not been observed during AT development or under pathological conditions. Multiple upstream signals, such as MST1/2 and MAP4K4, are involved in the regulation of LATS1/2^20^. Thus, manipulating the upstream components of the Hippo pathway may have more physiological or pathological relevance. In addition, released free fatty acid during obesity and mechanical cues have been reported to induce or activate YAP/TAZ^61,62^. Whether these regulations contribute to the development of AT fibrosis warrant future investigation.

In summary, we have uncovered a core mechanism underlying AT fibrosis by which the Hippo pathway orchestrates a cellular transition from adipocyte to myofibroblast when the pathway is inactivated, and a functional shift of adipocytes from energy storage to ECM remodeling upon its inhibition during obesity, both in close coordination with TGFβ signaling. These findings not only imply the Hippo pathway in the maintenance of adipocyte identity but also reveal a sophisticated cell crosstalk between adipocyte, macrophage and fibroblast that maintain AT homeostasis. Its dysregulation leads to AT maladaptation to chronic energy excess and eventually develop into AT fibrosis. Importantly, targeting the Hippo pathway both in adipocytes and preadipocytes may combat AT fibrosis and improve metabolic health.

## Methods

### Mouse models

*Lats1*^*f/f*^ (024941, *C57BL/6-129-CD1*), *Lats2*^*f/f*^ (027934, *C57BL/6-129*), *Yap1*^*f/f*^ (027929, *C57BL/6-129*), *ob/+* (000632), *ROSA*^*mT/mG*^ (007676), Rosa26-floxed STOP-Cas9-EGFP mice (026179), *EIIa*^*Cre*^ (003724), *Adipoq*^*Cre*^ (028020) and *Adipoq*^*CreERT2*^ (025124) mice were obtained from The Jackson Laboratories. *Taz*^*f/f*^ mice (*C57BL/6-129*) were made as below: a plasmid donor, containing two 34-bp loxP sites in homologous arms flanking the exon 2 of *Wwtr1* gene, was designed to generate a *Wwtr1* floxed allele by homologous recombination. Rosa26-floxed STOP-Cas9-EGFP mice were crossed with either *EIIa*^*Cre*^ or *Adipoq*^*Cre*^ to generate a mouse line with constitutive expression of CAS9 in all tissues or in adipocytes. Unless noted otherwise, mice are on C57BL/6 background. Mice were housed in temperature (22 ± 1 °C)- and humidity (60 ± 10%)-controlled rooms under a 12-h light–dark cycle, provided with chow diet (1010063, Xietong Shengwu) and water ad libitum, except for fasting experiments. All animal procedures were performed in compliance with protocols approved by the Institutional Animal Care and Use Committee of Peking University and conformed to the Guide for the Care and Use of Laboratory Animals.

For conditional deletion of *Lats1* and *Lats2*, tamoxifen (20 mg/ml in corn oil) (Sigma) was i.p. injected into 8-week-old male mice at 100 mg/kg for 3 doses every other day. For conditional deletion of YAP and TAZ, tamoxifen (20 mg/ml in corn oil) was i.p. injected into male mice fed a HFD (D12492, Research Diets) for 11 weeks at 50 mg/kg for 5 doses every day. For chemical injection experiments, VP (25 mg/kg) was dissolved in 2% DMSO, 30% PEG 300 and 2% Tween 80 (Vehicle). For insulin tolerance test experiments, male mice were fasted for 4 h, followed by i.p. injection with insulin (Eli Lilly, 1.5 U/kg). For glucose tolerance test experiments, male mice were fasted for 14 h, followed by i.p. injection with D-[+]-glucose (Sigma-Aldrich, 2 g/kg).

Body composition was measured by dual-energy X-ray absorptiometry (DXA) using a PIXImus densitometer (GE Lunar). Serum levels of NEFA were measured using LabAssay NEFA kit (Wako).

### Murine white adipocyte cell culture

For all the cellular assays, pooled scWAT from P10 to P21 WT mice were utilized to isolate SVF, which was cultured in growth media (DMEM containing 10% fetal bovine serum (FBS) and 1% penicillin/streptomycin. For adipogenic differentiation, nearly confluent cultures of SVF were treated with an adipogenesis induction cocktail (growth media supplemented with 0.9 μg/ml insulin, 1 nM T3, 0.5 μM dexamethasone, 0.5 mM 3-Isobutyl-1-methylxanthine, 125 μM indomethacin, 1 μM rosiglitazone) for 48 h. After 48 h, the cells were transferred to maintenance medium (4.5 μg/ml insulin, 1 nM T3). Medium was replaced every day, and cells were analyzed at the indicated days.

### BMDM isolation

Bone marrow derived macrophages (BMDMs) were isolated and differentiated as previously described^63^. In brief, the femurs and tibias were isolated from 8- to 12-week-old male C57BL/6 mice. After cutting off the ends of the bones and flushing with PBS, collect bone marrow using Ficoll-Paque™ PLUS Media (GE Healthcare) gradient. The macrophage precursors were differentiated in DMEM supplemented with 10% FBS, 10 ng/ml M-CSF and 1% penicillin/streptomycin at 37 °C in a humidified 5% CO_2_ incubator for 7 days. For inducing alternative activation, mature BMDMs were treated with both recombinant IL-4 (20 ng/ml) and IL-13 (20 ng/ml) for 24 h and harvested for subsequent analysis.

### HEK-293T cell culture

HEK-293T (ATCC, CRL-3216) were cultured in DMEM supplemented with 10% FBS and 1% penicillin/streptomycin. The cells were maintained at 37 °C in a humidified atmosphere with 5% CO_2_.

### RNA isolation and RT-qPCR

Total RNA was isolated using Trizol reagent (Sigma) according to the manufacturer’s instruction. RNA (0.1–1 μg) was reverse-transcribed to generate cDNA using the 5× All-In-One MasterMix kit (abm) and then subjected to RT-qPCR with ChamQ SYBR qPCR master mix (Vazyme) on a StepOnePlus Real-Time PCR System (Applied Biosystems). Relative expression level of mRNAs was calculated using the 2^(−ΔΔCT)^ method with 36B4 as an internal control. Primers used in this study are listed in Table S1.

### Construction of AAV-mediated CRISPR/Cas9 shuttle plasmids

*Tgfbr1 (T204D)* and *Tgfb1 (2CS)* cDNAs were cloned into pAAV-CAG-MCS-flag vector, which was modified from pAAV-CAG-GFP (from Edward Boyden; Addgene, 37825). *GFP*, *Yap (5SA)* and *Taz (4SA)* cDNAs were cloned into pAAV-ADP-MCS-flag vector (Fig. S3C). *Tgfbr1 (T204D)* cDNAs was cloned into pAAV-EF1A-DIO-GFP vector (from Karl Deisseroth; Addgene, 27056). Two Frt sites were cloned into the pAAV-EF1A-DIO- TGFβR1 (T204D) vector to generate pAAV-EF1A-FIO-TGFβR1 (T204D). FLPo, a codon-optimized version of FLP recombinase, was cloned into pAAV-ADP-MCS-flag vector^64,65^. Integrated with CRE-ERT2 system, the Frted Inverted Open reading frame (FIO) sequence was inverted via FLPo driven by adiponectin promoter, which achieved expression of TGFβR1 (T204D) specifically in mature adipocytes, avoiding the inhibitory effect of gene expression due to repressed adiponectin activity after *Lats1/2* deletion upon tamoxifen administration. To simultaneously knockout multiple genes, gRNA cassettes linked with 20 bp linker targeting different genes were assembled into pAAV-U6-sgRNA vector derived from pAAV-U6-sgRNA-CMV-GFP-HA vector (Addgene, 85451)^66^. Each gRNA sequence was firstly inserted into lentiCRISPR V2 vector (Addgene, 52961)^67^. Next, gRNA cassettes containing U6 promoter, gRNA and gRNA scaffold were subcloned into pAAV-U6-sgRNA-CMV-MCS-HA vector derived from pAAV-U6-sgRNA-CMV-GFP-HA vector. The genes of interest were cloned into pAAV-U6-sgRNA-CMV-MCS-HA vector to simultaneously knockout and overexpress genes. Sequences of gRNAs used in this study are listed in Table S2^68^.

### AAV production

The shuttle plasmids were co-transfected into HEK-293T (ATCC, CRL-3216) with pAAV-Helper and pAAV-RC2/8 plasmid (for in vivo experiments) or pAAV-DJ plasmid (for in vitro experiments). After co-transfection for 60 h, the cells were harvested and re-suspended in AAV lysis buffer (150 mM NaCl, 20 mM Tris pH 8.0). After repeated freezing and thawing, the virus particles were purified via discontinuous iodixanol gradient centrifugation. The viral titer was determined by RT-qPCR.

For AAV-mediated *lats1/lats2/yap/taz/tead*s knockout, viruses (10^12^ gene copy number (GC) per well of 12-well plate) were added to cells at the indicated days. For AAV-mediated gene overexpression, the viruses (2 × 10^11^ GC per fat pad) were administrated to 8-week-old male mice through local injection. For *Lats1*^*f/f*^*Lats2*^*f/f*^*ob/ob* or *Lats1*^*f/f*^*Lats2*^*f/f*^ mice fed a HFD for 16 weeks, 5 × 10^11^ GC per fat pad of AAV-ADP-GFP or AAV-ADP-Cre was used.

### Histology

Dissected tissues (BAT, scWAT, vWAT and Liver) were fixed in 4% paraformaldehyde overnight, dehydrated, and embedded in paraffin for sectioning. Paraffin sections (5 μm) were stained with hematoxylin and eosin, Masson’s trichrome (Leagene, DC0033), or picrosirius red (Leagene, DC0041) according to the manufacturers’ instructions. For F4/80 immunostaining, slides were incubated with anti-F4/80 (Cell signaling, 70076,1:200), followed by an incubation with biotin-conjugated goat anti-rabbit IgG (H+L) and streptavidin–HRP conjugate (ZSGB-Bio, 1:200). Images were captured on an OLYMPUS BX51 or OLYMPUS BX53 microscope.

### Immunofluorescence

Mouse ATs were embedded and cryo-frozen in Tissue-Tek O.C.T (Sakura, 4583). Cryosections (10 μm) were cut with a Cryostat (Leica, CM1950) and fixed in 4% paraformaldehyde for 30 min. After washing with PBS, the sections were blocked with 5% Bovine Serum Albumin (BSA) in PBS for 1 h at room temperature (RT). The sections were then incubated with the following primary antibodies at 4 °C overnight: anti-SMA (Cell Signaling, 19245, 1:300), anti-p-SMAD2 (S465/S467) (Cell Signaling, 18338, 1:100), anti-Col1a1 (Cell Signaling, 72026, 1:100). After washing with PBST, sections were incubated with the following primary or secondary antibodies for 2 h at RT: APC-conjugated anti-CD26 (DPP4) (Biolegend, 137807, 1:100), eFluor 660-conjugated anti-Ki67 (eBioscience, 50-5698-82, 1:200), Alexa Fluor 647-conjugated anti-rabbit IgG (Thermo, A21244, 1:300), PE-conjugated anti-rabbit IgG (Thermo, 12-4739-81, 1:400). Finally, the sections were incubated with 4′6′-diamidino-2-phenylindole (DAPI) for 10 min at RT. Images were captured on a Carl ZEISS LSM 710 NLO, Nikon AIR-si or EVOS® FL (Life Technologies) microscope. Image quantification was performed using the ImageJ software.

For immunolabeling of cultured cells, the isolated cells were seeded on cell culture dish, and fixed wih 4% paraformaldehyde for 20 min at RT. Cells were permeabilized with 0.05% saponin (Sigma, S7900) for 10 min and then blocked with 5% BSA for 1 h at RT. After washing with PBST, cells were incubated with primary antibodies at 4 °C overnight. After washing, cells were treated with secondary antibodies or primary antibodies conjugated with different fluorescent dyes at RT for 2 h and counterstained with DAPI.

### Immunoblotting

Cells or frozen tissue samples were lysed in modified RIPA buffer [450 mM NaCl, 1% NP-40, 0.1% SDS, 0.5% Deoxycholic acid (sodium salt), 50 mM Tris pH 7.5, and cocktail protease inhibitors]. Proteins were separated by SDS-PAGE and transferred to nitrocellulose membrane following standard protocols. Membranes were blotted with primary antibodies against HSP90 (Santa Cruz, sc-13119, 1:10,000), α-Tubulin (Sigma, T6199, 1:10,000), Lamin B1 (Proteintech, 66095-1-Ig, 1:10,000), LATS1 (Cell Signaling, 3477, 1:1000), LATS2 (Bethyl Laboratories, A300-479A, 1:1000), YAP (Cell Signaling, 4912 or 14074, 1:1000), TAZ (Cell Signaling, 4883 or 83669, 1:1000), p-YAP (Ser 112) (Cell Signaling, 4911), p-TAZ (Ser 89) (Cell Signaling, 59971, 1:1000), HSL (Cell Signaling, 4107, 1:1000), Perilipin 1 (Vala Sciences, 4854, 1:10,000), SMAD2/3 (Cell Signaling, 8685, 1:1000), p-SMAD2 (Ser465/467)/SMAD3 (Ser423/425) (Cell Signaling, 8828, 1:1000), αSMA (Cell Signaling, 19245, 1:5000), FLAG (Abmart, M20008, 1:5000), Caspase 3 (Cell Signaling, 9662,1:1000), MST1 (Cell Signaling, 3682, 1:1000), MST2 (Cell Signaling, 3952, 1:1000), p-SAPK/JNK (Thr183/Tyr185) (Cell Signaling, 4668, 1:1000), SAPK/JNK (Cell Signaling, 9252, 1:1000), p-AKT (Ser473) (Cell Signaling, 4060, 1:1000), AKT (Cell Signaling, 9272, 1:1000), p-p38 MAPK (Thr180/Tyr182) (Cell Signaling, 4511, 1:1000), p38 MAPK (Cell Signaling, 8690, 1:1000), p-MLC2 (Ser19) (Cell Signaling, 3671, 1:1000), MLC2 (Proteintech, 10906-1-AP, 1:1000), ERK1/ERK2 (ABclonal, A10613, 1:1000), p-ERK1(T202/Y204)/ERK2(T185/Y187) (ABclonal, AP0472, 1:1000), GFP (Thermo Scientific, A-11120, 1:1000), Myc (Cell Signaling, 2276, 1:2000) and Col1a1 (Cell Signaling, 72026, 1:1000) followed by incubation with HRP-conjugated anti-rabbit secondary antibody (Thermo Scientific, 31460, 1:10,000) or anti-mouse secondary antibody (Thermo Scientific, 32430, 1:10,000). Nuclear and cytoplasmic fractionation were performed using the Nuclear and Cytoplasmic Protein Extraction Kit (Beyotime, P0028), according to the manufacturer’s manual. 7.5% phos-tag gels (APExBIO) were used to detect TAZ phosphorylation levels. Super ECL Detection Reagent (Yeasen) were used to visualize blots in an automatic chemiluminescence analysis system (Tanon). All quantifications of protein expression were performed using ImageJ software. The uncropped and unprocessed scans of the blots were provided in a Source data file.

### Immunoprecipitation (IP)

For IP assay, cells were harvested and lysed on rotator for 2 h at 4 °C using IP buffer (10% Glycerol, 1% NP-40, 5 mM EDTA, 120 mM NaCl, 50 mM Tris-HCl, pH7.5) with cocktail protease inhibitors. Supernatant was collected after centrifugation at 12,000 × *g* for 15 min. Ten percent of supernatant was used for input fraction and the rest was immunoprecipitated by anti-HA-magnetic beads (Thermo Scientific, 88836) for 2 h at 4 °C. The protein containing HA tag was collected through magnetic enrichment, whereas protein without HA tag was excluded after washing with IP buffer for five times. The protein containing HA tag was separated from beads after incubation with 100 μl glycine solution (0.1 M, pH 2.0) for 10 min. Supernatant after magnetic enrichment was neutralized with 15 μl Tris solution (1 M, pH 8.0) and then prepared for IP assay.

### Isolation of stromal vascular fraction (SVF) and adipocytes

Inguinal scWAT were minced with scissors and digested with Collagenase Type I (180 U/ml, Worthington, LS004216) in SVF buffer [1.1 mM CaCl_2_, 2.7 mM KCl, 118 mM NaCl, 0.5 mM MgCl_2_, 0.4 mM NaH_2_PO4, 20 mM HEPES, 5.5 mM Glucose, 1% BSA (fatty-acid free)] at 37 °C with agitation for 50 min. The digested cell suspension was centrifuged at 520 × *g* for 5 min, resuspended in SVF buffer and passed through a 40-μm strainer. Floated cells were collected as adipocytes, and pelleted cells were resuspended in red blood cell lysis buffer (0.15 M NH_4_Cl, 10 mM NaHCO_3_, 1.1 mM EDTA) for 5 min at RT and then quenched in SVF buffer. Next, cells were collected by centrifugation at 520 × *g* for 5 min and recovered in FACS buffer (PBS containing 2% FBS and 1 mM EDTA).

### Flow cytometry

SVF isolated from scWAT was incubated with Fixable Viability Dye eFluor™ 780 (eBioscience, 65-0865-14) at RT for 15 min for live-dead staining and then centrifuged at 520 × *g* for 5 min. Pelleted cells were resuspended in FACS buffer containing anti-mouse CD16/CD32 Fc Block (Biolegend, 101302, 1:1000) for 5 min and then incubated with the following antibodies for 30 min at 4 °C in the dark: anti-mouse CD26 (DPP4)-allophycocyanin (APC) (Biolegend, 137807, 1:100), anti-mouse CD45-PerCP/Cy5.5 (Biolegend, 103131, 1:1000), anti-mouse CD31-PerCP/Cy5.5 (Biolegend, 102419, 1:300), F4/80-PE (Cell Signaling, 64763, 1:80), CD45-AF700 (BD Biosciences, 560510, 1:800), CD206-PE-Cy7 (Biolegend, 141720, 1:100), CD11c-AF647 (Biolegend, 117312, 1:100). After incubation, the cells were washed once with PBS and then resuspended in FACS buffer. The cells were sorted using a BD FACSAria III cytometer (BD Biosciences) or analyzed using a CytoFLEX S flow cytometer (Beckman Coulter). Flow cytometry plots were analyzed with the FlowJo (v10) or CytExpert (v2.3.0.84) software.

### Magnetic activated cell sorting

SVF isolated from scWAT was incubated with anti-F4/80-Biotin antibody (Miltenyi Biotech., 130-116-514, 1:100) for 20 min in the dark on ice, followed by washing with 2 ml MACS buffer (PBS containing 0.5% BSA and 2 mM EDTA) and centrifuged at 520 × *g* for 5 min. Pelleted cells were resuspended in 100 μl MACS buffer containing 10 µl of Streptavidin MicroBeads (Miltenyi Biotech.) for 20 min. After washing once with MACS buffer and resuspending in 500 µl MACS buffer, cell suspension was placed onto the pre-wet LS Column in the magnetic field of a MACS Separator (Miltenyi Biotech.). Unlabeled cells that pass through the column by washing column 3 times with 9 ml MACS buffer were collected as the SVF F4/80^−^ cell fraction. After removing column from the separator, 5 ml MACS buffer was added onto the column. Magnetically labeled F4/80^+^ cells were flushed out to a collection tube by firmly pushing the plunger into the column and collected by centrifugation at 520 × *g* for 5 min. scWAT from three male mice were pooled as one sample.

### Transplantation

Cell transplantation was performed as previously described with minor modifications^41^. Briefly, GFP^+^DPP4^+^ cells were purified by FACS from the scWAT of 6–10 pooled P21-P35 L1L2-AKO^LSL-CAS9-GFP^ or L1L2-AKO^mTmG^ as donor cells. GFP^+^DPP4^+^ donor cells were collected by centrifugation to ~3000 cells/ml and mixed 1:1 with Matrigel (phenol free and growth factor reduced; Corning) on ice. L1L2-AKO mouse pups at the age of 7–10 days were anesthetized using an isoflurane nose cone, and abdominal hair was removed before the creation of an incision to expose the bilateral inguinal fat pads. Twelve microliters of the donor cell-Matrigel mixture was injected along the edge of the inguinal fat pad. The recipient animals were closed with a 6-0 polypropylene suture and placed back with their litters. After the indicated time, donor cells were harvested from the recipient animals by dissection of the entire inguinal fat pad and analyzed through flow cytometry. Biological replicates here mean separate pools of donor cells transplanted into separate individual recipient mice.

### RNA-sequencing analysis

Adipocytes were isolated from four pooled male P7 L1L2-FF or L1L2-AKO mice. Inguinal scWAT were minced carefully with scissors for 3–5 min and digested with 4 mg/ml Collagenase Type I (in 4 ml PBS containing 1% BSA) at 37 °C with agitation for 12–15 min. The digested cell suspension was quenched in 4 ml PBS containing 1% BSA, followed by free floating for 5 min. After removing infranatant with a syringe and needle, floated cells were filtered using a 300 μm strainer and washed with 3 ml PBS containing 1% BSA. Floated adipocytes were collected by centrifugation at 30 × *g* for 5 min and lysed with Trizol reagent for total RNA extraction.

RNA-seq libraries were generated with three biological replicates. High-throughput sequencing was performed as pair-end sequencing using BGISEQ-500 (Beijing Genomics institution). The quality of the reads was assessed using the FastQC (v0.11.9) tool. Reads were aligned to a mouse genome (mm10) using HISAT2 (v2.1.0). Bam files were sorted and indexed in SAMtools (v1.3.1), followed by analyzing with StringTie (v1.3.5). Visualization was performed within the R/Bioconductor environment (v3.6.3).

### Statistics and reproducibility

Data were presented as means ± SEM. The *n* value means biological replicates with individual values representing a mouse, unless otherwise indicated. For correlation analysis in clinical cohorts from GEO database, linear regression analysis was performed. Statistical analyses were performed using GraphPad Prism 8.0 (GraphPad Software, Inc., La Jolla, CA, USA) or Microsoft Excel 2019. For comparisons between two independent groups, two-tailed unpaired Student’s *t* test was used. Statistical significance of differences between multiple groups was determined using one-way or two-way ANOVA with Bonferroni’s multiple-comparisons test. *P* < 0.05 was considered statistically significant. *, **, and *** correspond to *P* values of <0.05, <0.01, and <0.001, respectively.

### Reporting summary

Further information on research design is available in the Nature Research Reporting Summary linked to this article.

## Supplementary information

Supplementary Information

Reporting Summary

## Data Availability

Our RNA-seq data generated in this study have been deposited in the NCBI Gene Expression Omnibus repository under accession code GSE184826. The RNA-seq data used in this study are publicly available in the NCBI Gene Expression Omnibus repository under the accession numbers GSE141432 and GSE152991. Gene list involved in Hippo pathway was from Hallmark gene sets from molecular signatures database v7.5.1 (https://www.gsea-msigdb.org/gsea/msigdb/index.jsp). Source data are provided with this paper.

## Source data

Source Data File
