# Supplementary material for: The Hippo pathway links adipocyte plasticity to adipose tissue fibrosis
Source: Nat Commun. 2022 Oct 13;13:6030. doi: 10.1038/s41467-022-33800-0 (PMC9562301; doi:10.1038/s41467-022-33800-0)
Supplement: Supplementary file 1 — Supplementary Information [file 41467_2022_33800_MOESM1_ESM.pdf]

Supplementary Figure 1

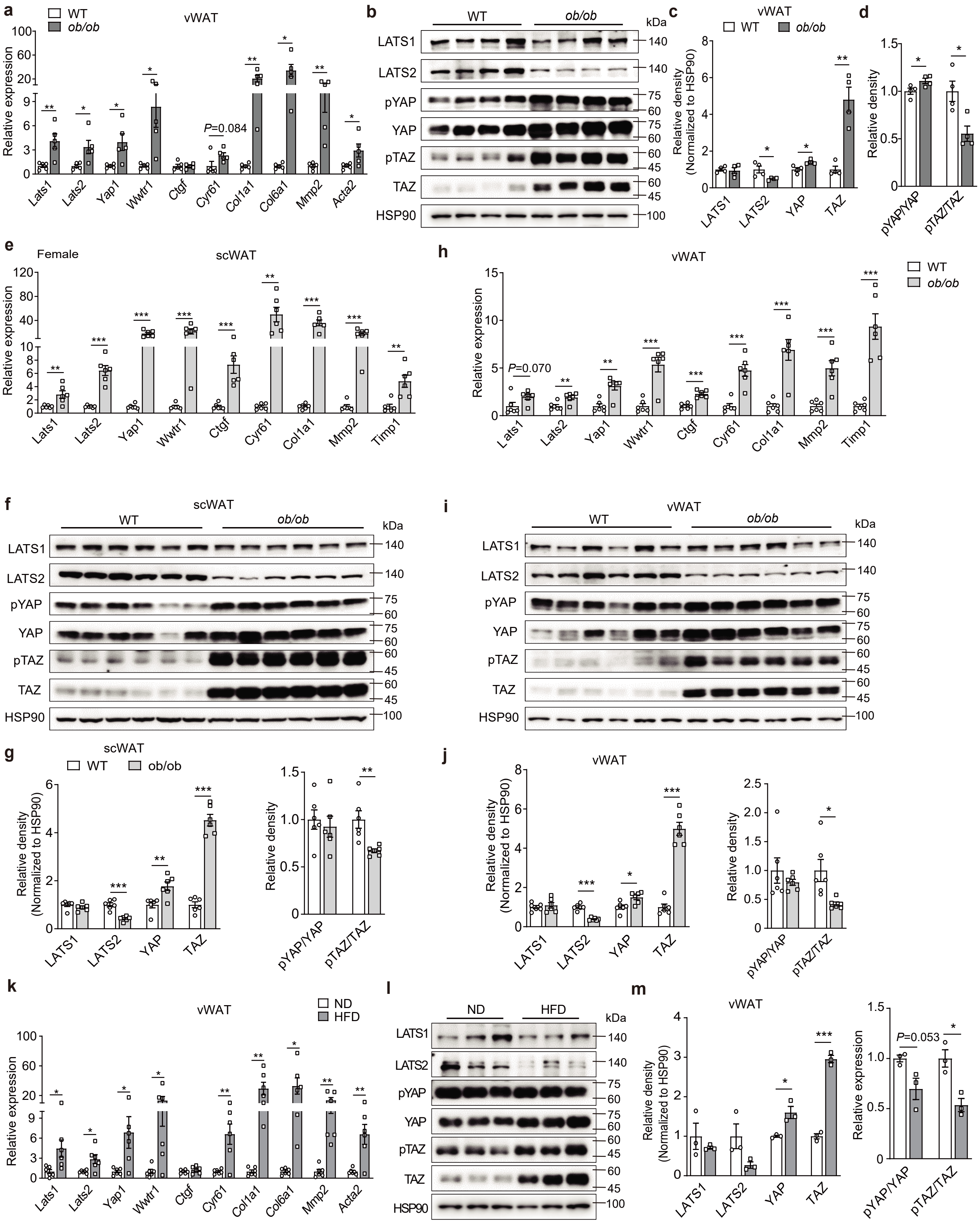

Supplementary Figure 1. Hippo pathway is inactivated in obesity-induced AT fibrosis.

a-d, Male WT or *ob/ob* mice were analyzed at 12 weeks old. **a**, RT-qPCR analysis of genes involved in Hippo pathway and fibrotic response in vWAT (n=5 mice). **b**, Immunoblot analysis of LATS1/2, YAP/TAZ, p-YAP and p-TAZ in vWAT. **c**, Quantification of protein expression from vWAT shown in (**b**) (n=4 mice). **d**, Quantification of phosphorylation level of YAP/TAZ (n=4 mice). **e-j**, Female WT or *ob/ob* mice were analyzed at 12 weeks old. **e**, RT-qPCR analysis of genes involved in Hippo pathway and fibrotic response in (**e**) scWAT or (**h**) vWAT (n=6 mice). **f**, Immunoblot analysis of LATS1/2, YAP/TAZ, p-YAP and p-TAZ in (**f**) scWAT or (**i**) vWAT, respectively. **g**, Quantification of protein expression from (**g**) scWAT or (**j**) vWAT shown in (**f**) and (**i**) (n=6 mice). **k**, RT-qPCR analysis of genes involved in Hippo pathway and fibrotic response in vWAT (n=6) obtained from male mice fed a HFD or ND for 18 weeks. **l**, Immunoblot analysis of LATS1/2, YAP/TAZ, p-YAP and p-TAZ in vWAT of ND and HFD mice (n=3 mice). **m**, Left, quantification of protein expression from scWAT or vWAT shown in (**l**); right, quantification of phosphorylation level of YAP/TAZ (n=3 mice). Data are means ± SEM. Two-tailed unpaired student's *t*-test; *P < 0.05, **P < 0.01, ***P < 0.001. Exact P values are provided in a Source Data file.

Supplementary Figure 2

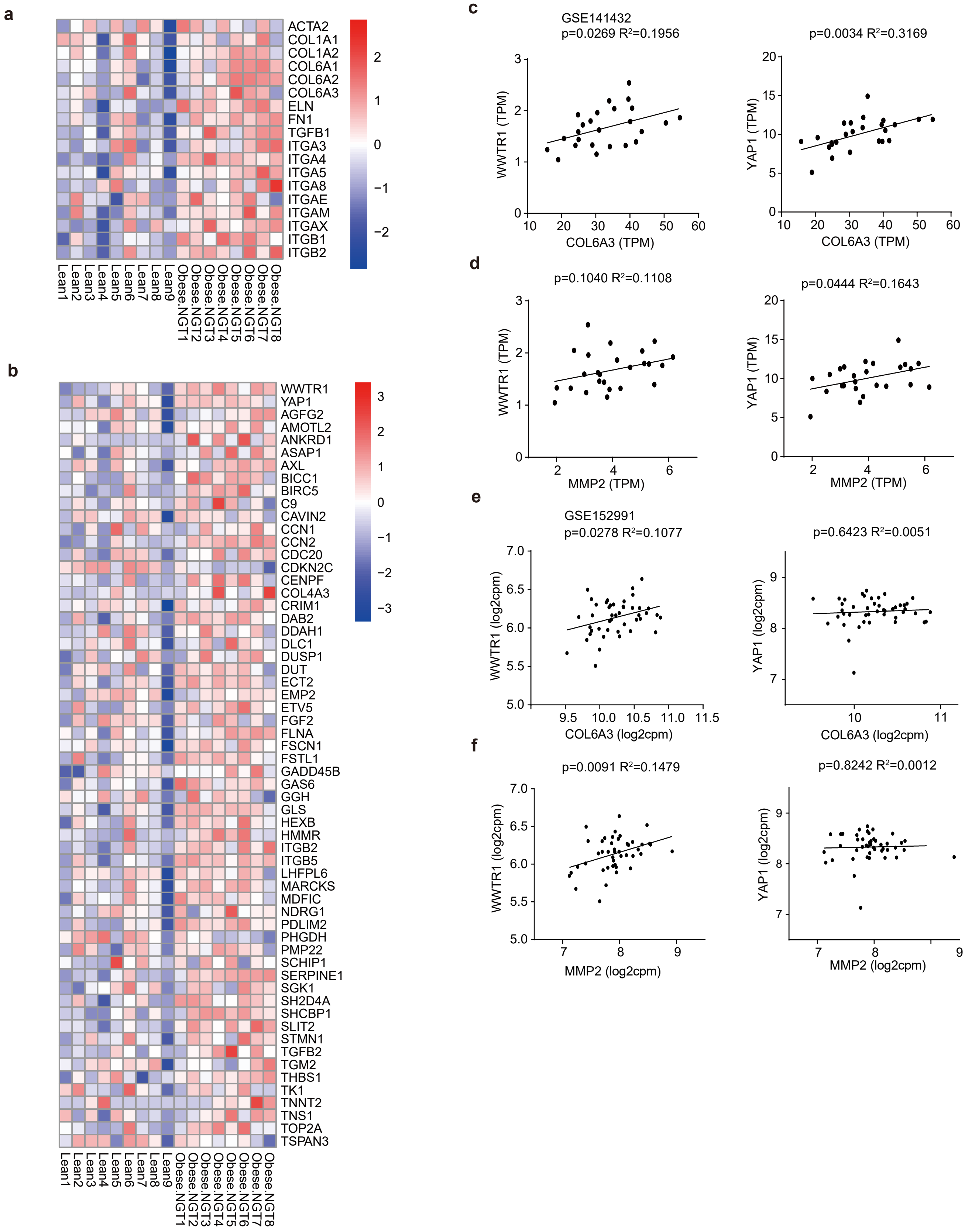

Supplementary Figure 2. Genes involved in Hippo pathway and in AT fibrosis are positively correlated in humans.

a, **b**, Heatmap analysis of genes involved in ECM remodeling (**a**) and Hippo pathway (**b**, molecular signatures database: cordenonsi_YAP_conserved_signiature) extracted from the RNAseq data (TPM values) of abdominal scWAT of 9 healthy lean individuals (Lean) and 8 obese normal glucose tolerance subjects (Obese NGT) (Gene Expression Omnibus (GEO) repository, accession number GSE141432). **c**-**f**, Correlation analysis between genes involved in Hippo pathway and ECM remodeling, using publicly available transcriptomic data. **c**, **d**, RNAseq of scWAT of 25 subjects (GSE141432). **e**, **f**, RNAseq of scWAT of 45 subjects (GSE152991). TPM, transcripts per million. CPM, read counts per million. Linear regression analysis (c-f); significance was calculated by two-tailed unpaired student's *t* test.

Supplementary Figure 3

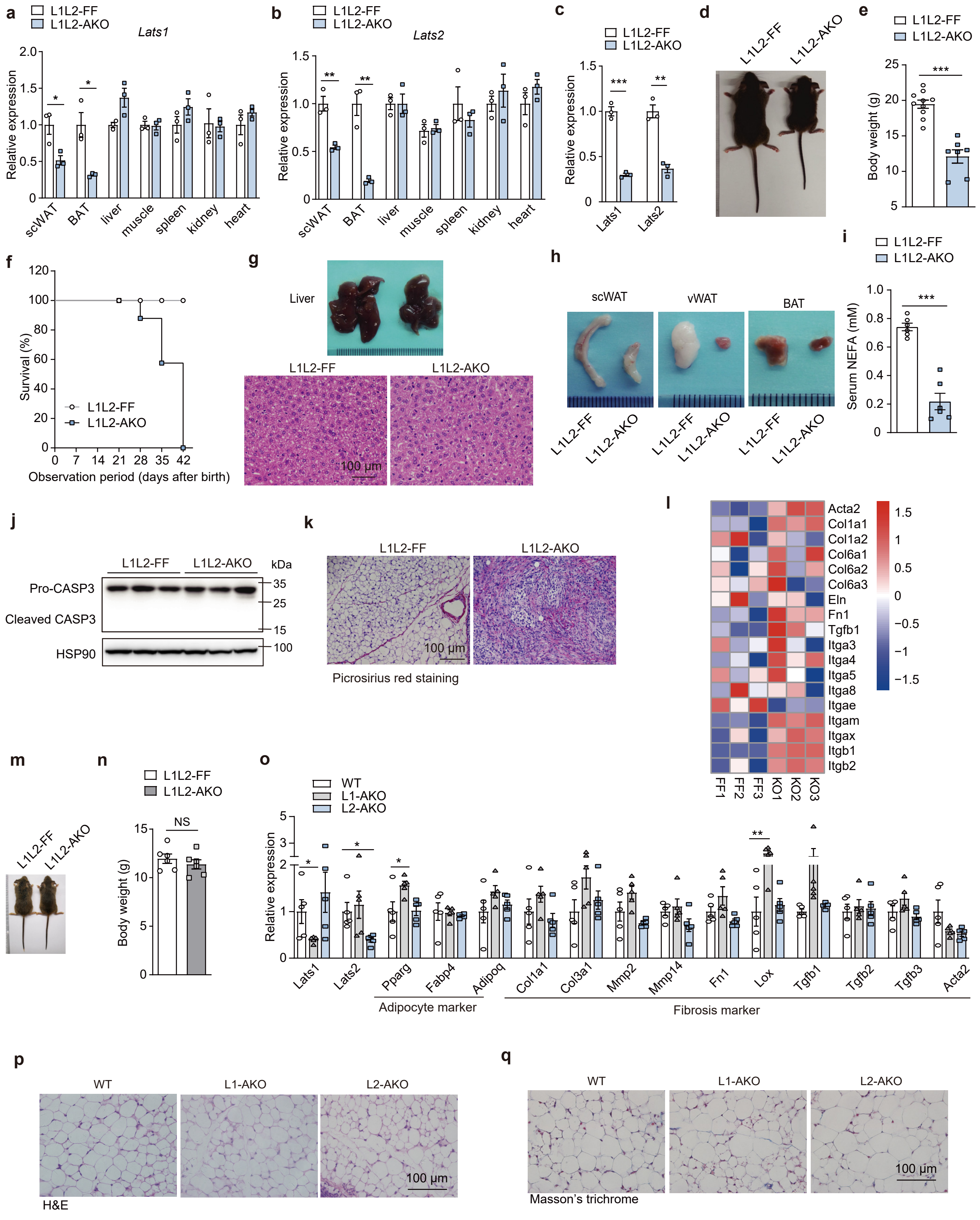

Supplementary Figure 3. Characterization of *Lats1/2*-deficient mice.

a, **b**, mRNA expression of *Lats1* (**a**) and *Lats2* (**b**) in scWAT, BAT, liver, muscle, spleen, kidney and heart in male P7 L1L2-FF and L1L2-AKO mice (n=3). **c**, mRNA expression of *Lats1* and *Lats2* in mature adipocytes isolated from P7 L1L2-FF or L1L2-AKO scWAT (n=3). Three male mice were pooled as one sample. **d-k**, Characterization of 5-week-old male L1L2-AKO and L1L2-FF mice. **d**, Gross appearance of mice. **e**, Quantification of body weight L1L2-AKO (n=7) and L1L2-FF (n=10) mice. **f**, Survival analysis (n=19). **g**, Representative liver sections with H&E staining. **h**, Representative images of scWAT, vWAT, BAT. **i**, Plasma non-esterified free fatty acids (NEFA) levels (n=6 mice). **j**, Immunoblot analysis of Pro-Caspase 3 (Pro-CASP3) and Cleaved Caspase 3 (CASP3) in scWAT (n=3 mice). **k**, Representative scWAT sections with Picrosirius red staining. Independent experiments were performed twice with similar results. **l**, ECM remodeling genes extracted from the RNAseq data (TPM values) of scWAT adipocytes of P7 L1L2-FF (FF) and L1L2-AKO (KO) mice. **m**, **n**, Body size (**m**) and body weight (**n**) of P21 male L1L2-FF and L1L2-AKO mice (n=6). **o**, mRNA expression of *Lats1/Lats2*, adipocyte markers and fibrotic markers of scWAT in 5-week-old male L1-AKO or L2-AKO mice (n=5). **p**, **q**, Representative sections of scWAT with Masson's trichrome (**p**) or H&E staining (**q**). Independent experiments were performed twice with similar results. Data are means ± SEM. Two-tailed unpaired student's *t*-test in (**a-c**), (**e**), (**i**), (**n**); One-way ANOVA with Bonferroni's multiple-comparisons test in (**o**); *$P < 0.05$, **$P < 0.01$, ***$P < 0.001$; NS, not significant. Exact *P* values are provided in a Source Data file.

Supplementary Figure 4

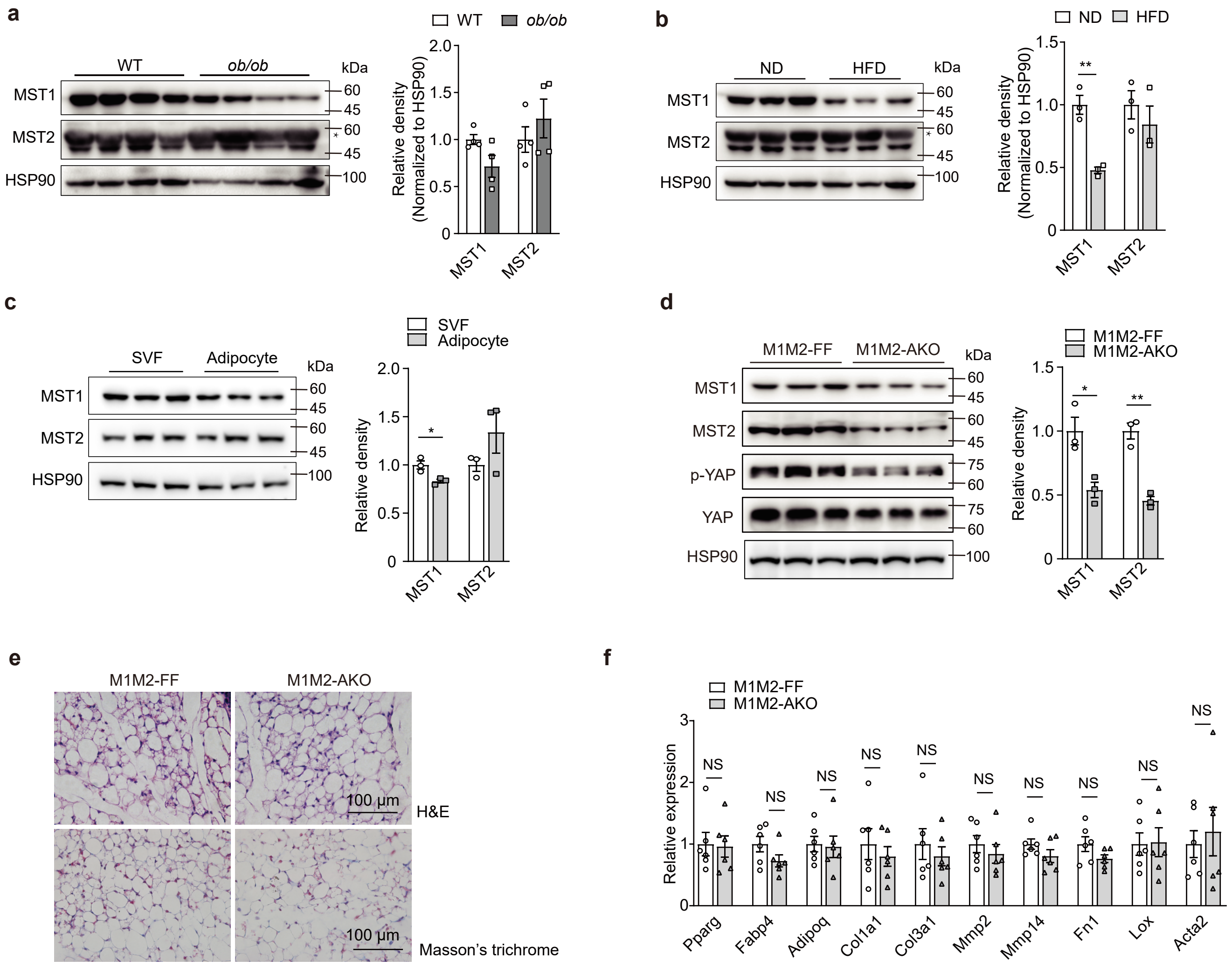

Supplementary Figure 4. ***Lats1/2*** **deficiency-induced AT fibrosis does not depend on MST1/2.**

a, **b**, Immunoblot analysis of MST1 and MST2 in 12-week-old male *ob/ob* mice (**a**, n=4) and male mice fed a HFD for 18 weeks (**b**, n=3). Right, quantification of protein levels of MST1/2. The asterisks indicated non-specific bands. **c**, Immunoblot analysis of protein expression of MST1 and MST2 in scWAT adipocytes and SVF isolated from 5-week-old male WT mice. Right, quantification of relative protein expression of MST1 and MST2 (n=3 mice). **d**, Immunoblot analysis of the indicated protein in scWAT of 5-week-old male M1M2-FF and M1M2-AKO mice. Right, quantification of the indicated protein expression (n=3). **e**, Representative sections of scWAT with H&E or Masson's trichrome staining. Independent experiments were performed twice with similar results. **f**, RT-qPCR for adipocyte and fibrotic marker gene expression in scWAT (n=6 mice). Data are means ± SEM. Two-tailed unpaired student's t-test; *$P < 0.05$, **$P < 0.01$, ***$P < 0.001$; NS, not significant. Exact P values are provided in a Source Data file.

Supplementary Figure 5

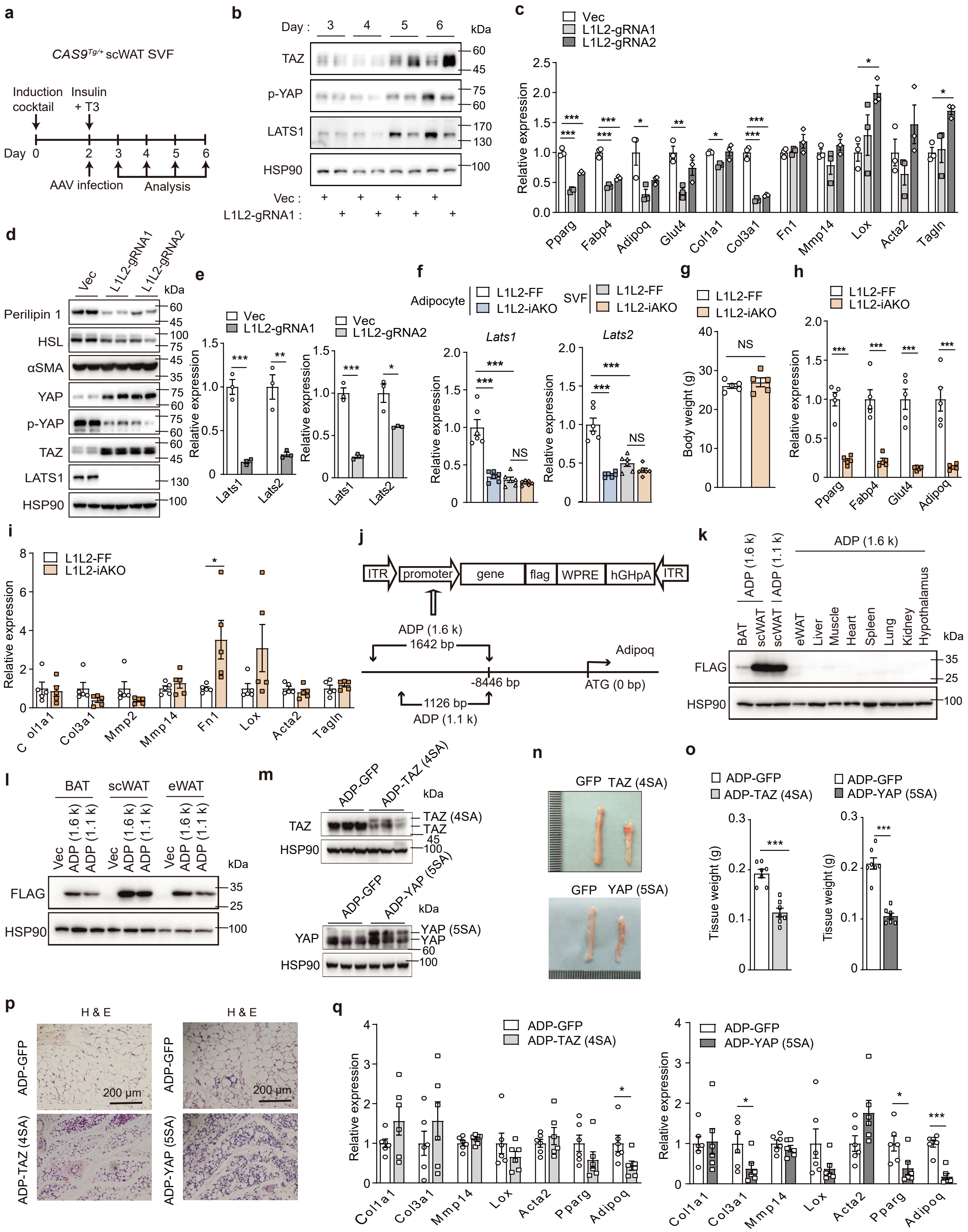

Supplementary Figure 5. LATS1/2 maintain adipocyte identity in a cell-autonomous manner.

a, Schematic overview of AAV infection of *Cas9*$^{Tg/+}$ SVF undergoing differentiation. **b**, Immunoblot analysis of time-course expression of TAZ, p-YAP, and LATS1 in Vec or LATS1/2-gRNA (L1L2-gRNA1) transduced cells. Independent experiments were performed three times with similar results. **c**, RT-qPCR analysis of adipocyte identity and fibrosis markers of differentiated adipocytes transduced with Vec, L1L2-gRNA1 and L1L2-gRNA2 on day 2 post differentiation, and then analyzed 5 days later (n=3 biologically independent cell cultures). **d**, Immunoblot analysis of protein expression of adipocyte identity, fibrosis markers and Hippo signal proteins of cells in (**c**). **e**, Knock-down efficiency of L1L2-gRNA1 (left) and L1L2-gRNA2 (right) in differentiated adipocytes transduced with the indicated gRNAs (n=3 biologically independent cell cultures). **f**, mRNA expression of *Lats1* and *Lats2* in adipocyte and SVF from eight-week-old male L1L2-FF and L1L2-iAKO mice that were i.p. administered with a dose of tamoxifen (100 mg/kg) and then analyzed 6 days later (n=6 mice). **g-i**, Body weight (**g**), mRNA expression of adipocyte identity (**h**) and fibrosis markers (**i**) from eight-week-old male L1L2-FF and L1L2-iAKO mice that were i.p. administered with 3 doses of tamoxifen (100 mg/kg) every other day and then analyzed 4 weeks later (n=5 mice). **j**, The structure of the AAV-*Adiponectin* promoter (ADP) vector. **k**, Immunoblot analysis of AAV-ADP-GFP-Flag expression at the indicated tissues of WT mice whose scWAT were locally injected with the viruses. Independent experiments were performed twice with similar results. **l**, Comparison of FLAG expression levels driven by ADP (1.6 k) or ADP (1.1 k) promoter. Independent experiments were performed twice with similar results. **m-q**, Eight-week-old male mice injected with AAV-ADP-GFP, AAV-ADP-TAZ (4SA) or AAV-ADP-YAP (5SA) in

scWAT for 4 weeks. **m**, Immunoblot analysis of protein expression of TAZ(4SA) or YAP(5SA) in scWAT. **n**, Representative images of scWAT injected with AAV-ADP-GFP, AAV-ADP-TAZ (4SA) or AAV-ADP-YAP (5SA). **o**, Quantification of scWAT weight (n=7 mice). **p**, Representative scWAT sections with H&E staining. Independent experiments were performed twice with similar results. **q**, RT-qPCR analysis of fibrosis and adipocyte markers (n=6 mice). Data are means ± SEM. Two-tailed unpaired student's *t*-test in (**e**), (**g-i**), (**o**), (**q**); One-way ANOVA with Bonferroni's multiple-comparisons test in (**c**); Two-way ANOVA with Bonferroni's multiple-comparisons test in (**f**); $^*P < 0.05$, $^{**}P < 0.01$, $^{***}P < 0.001$; NS, not significant. Exact *P* values are provided in a Source Data file.

Supplementary Figure 6

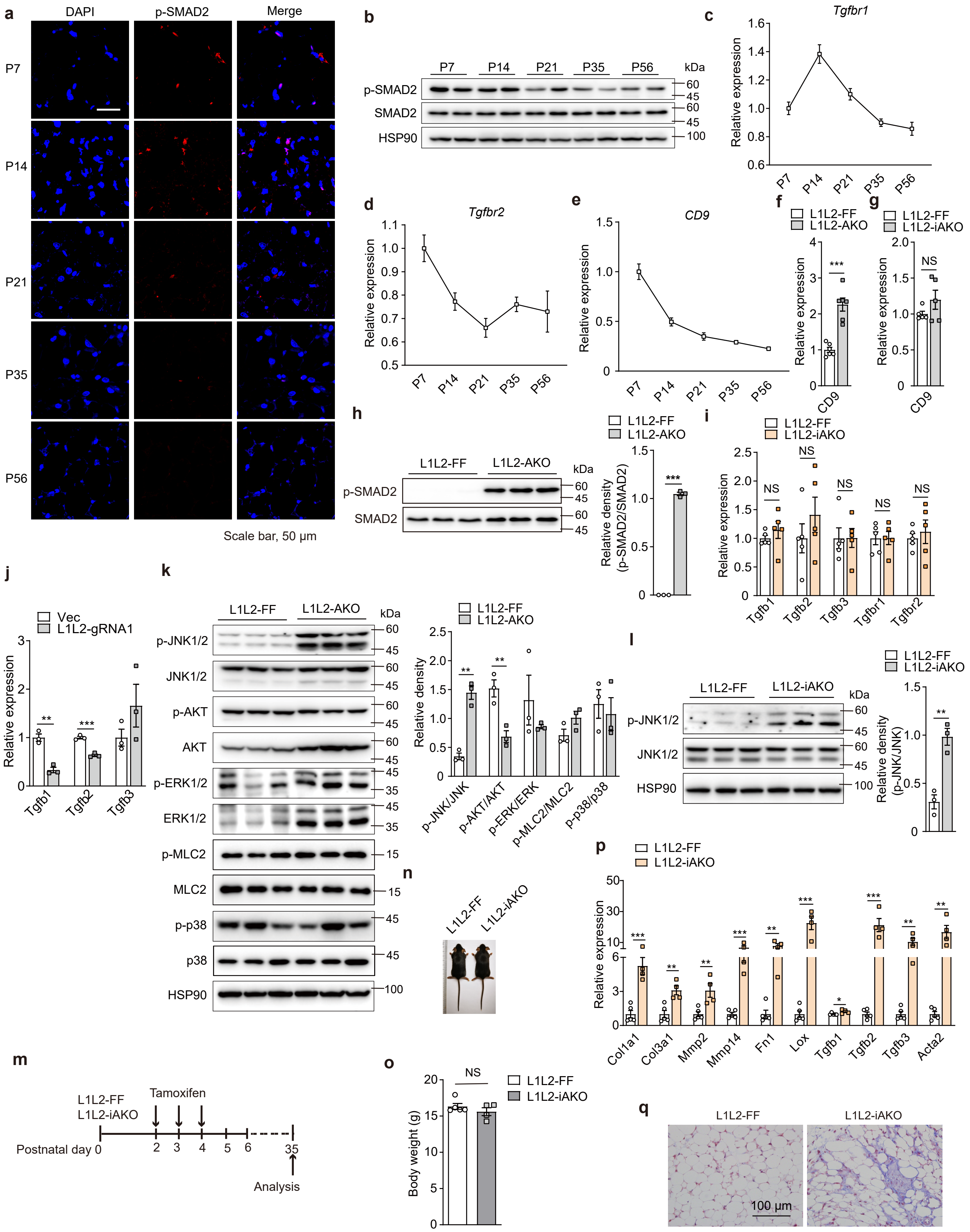

Supplementary Figure 6. TGFβ signaling may contribute to *Lats1/2*-deficiency induced fibrosis.

a, Representative sections of scWAT stained for p-SMAD2 (red) and nucleus (DAPI, blue) during growth. Independent experiments were performed twice with similar results. **b**, Representative immunoblot analysis of phosphorylation levels of SMAD2 in scWAT during growth. Independent experiments were performed twice with similar results. **c-e**, mRNA expression of scWAT *Tgfbr1 (c)*, *Tgfbr2 (d)* or *CD9 (e)* during growth (n=6 mice). **f**, **g**, mRNA expression of scWAT *CD9* in 5-week-old L1L2-AKO (**f**, n=6 mice) or in L1L2-iAKO mice as indicated in supplementary Figure 5g (**g**, n=5 mice). **h**, Immunoblot analysis (left) and quantification (right) of the p-SMAD2 in scWAT of male L1L2-FF or L1L2-AKO mice at five weeks old (n=3 mice). **i**, mRNA expression of scWAT *Tgfb1/2/3, Tgfbr1* and *Tgfbr2* of L1L2-FF or L1L2-iAKO mice as indicated in supplementary Figure 5g (n=5). **j**, mRNA expression of *Tgfb1/2/3* of differentiated adipocytes transduced with Vec or L1L2-gRNA1 AAV (n=3 biologically independent cell cultures). **k**, Immunoblot analysis (left) and quantification (right) of the non-canonical TGFβ pathways in scWAT of male L1L2-FF or L1L2-AKO mice at five weeks old (n=3 mice). **l**, Immunoblot analysis (left) and quantification (right) of phosphorylation levels of JNK in scWAT of male L1L2-FF or L1L2-iAKO mice four weeks after tamoxifen administration (n=3 mice). **m-q**, Three consecutive intragastric injections of tamoxifen (1.5 mg/mL) were given to newborn male L1L2-FF or L1L2-iAKO mice at P2–P3–P4 and all mice were analyzed at 5 weeks old. **n**, Gross pictures of mice. **o, p**, Body weight (**o**) or mRNA expression of fibrosis markers (**p**) of L1L2-FF (n=5) and L1L2-iAKO (n=4) mice. **q**, Representative Masson's trichrome staining of scWAT sections of L1L2-FF and L1L2-iAKO mice. Data are means ± SEM. Two-

tailed unpaired student's *t*-test in (**f**), (**g**), (**h-l**), (**o**); $*P < 0.05$, $**P < 0.01$, $***P < 0.001$; NS, not significant. Exact *P* values are provided in a Source Data file.

Supplementary Figure 7

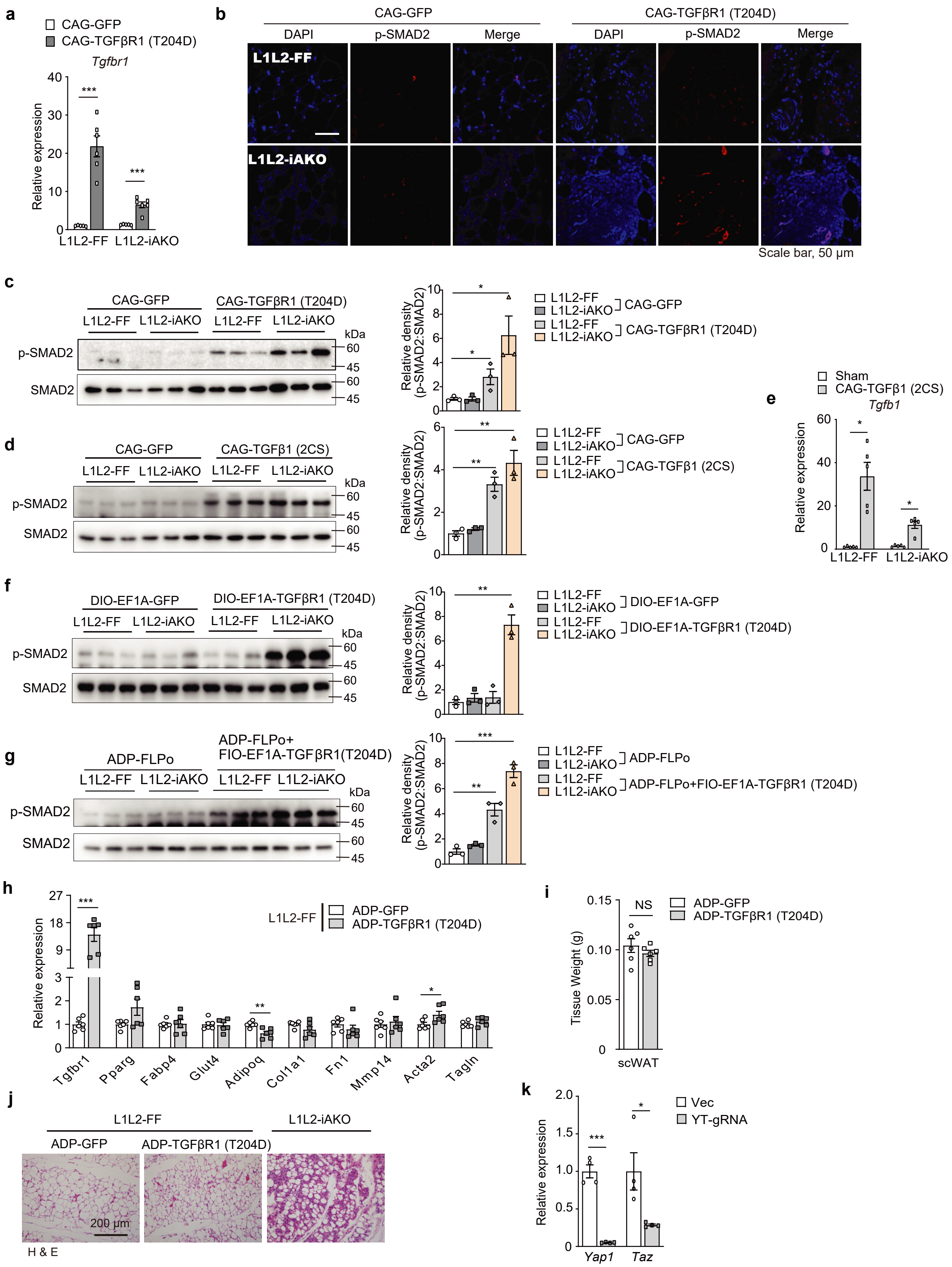

Supplementary Figure 7. Hippo pathway inactivation or TGFβ stimulation alone is not sufficient to induce AT fibrosis in adult mice.

a, Overexpression efficiency of AAV-CAG-TGFβR1 (T204D) in L1L2-FF or L1L2-iAKO scWAT transduced with AAV-CAG-GFP (n=5 mice) or AAV-CAG-TGFβR1 (T204D) (n=6 mice) followed by tamoxifen administration. **B**, Representative sections of indicated scWAT stained for p-SMAD2 (red) and nucleus (DAPI, blue). **c**, **d**, **f**, **g**, Left, immunoblot analysis of phosphorylation of SMAD2 (p-SMAD2) of the indicated mice; Right, quantification of relative phosphorylation levels of SMAD2 (n=3 mice). **e**, Overexpression efficiency of AAV-CAG-TGFβ1 (2CS) in scWAT (n=5 mice). **h-j**, Eight-week-old WT mice were subcutaneously injected with AAV-ADP-GFP or AAV-ADP-TGFβR1 (T204D). **h**, mRNA expression of fibrosis and adipocyte markers (n=6 mice). **I**, scWAT weight (n=6 mice). **j**, Representative scWAT sections with H&E staining. **k**, Knockdown efficiency of YT-gRNA in $Cas9^{Tg/+}$ SVF (n=4 biologically independent cell cultures). Data are means ± SEM. Two-tailed unpaired student's *t*-test; *$P < 0.05$, **$P < 0.01$, ***$P < 0.001$; NS, not significant. Exact *P* values are provided in a Source Data file.

Supplementary Figure 8

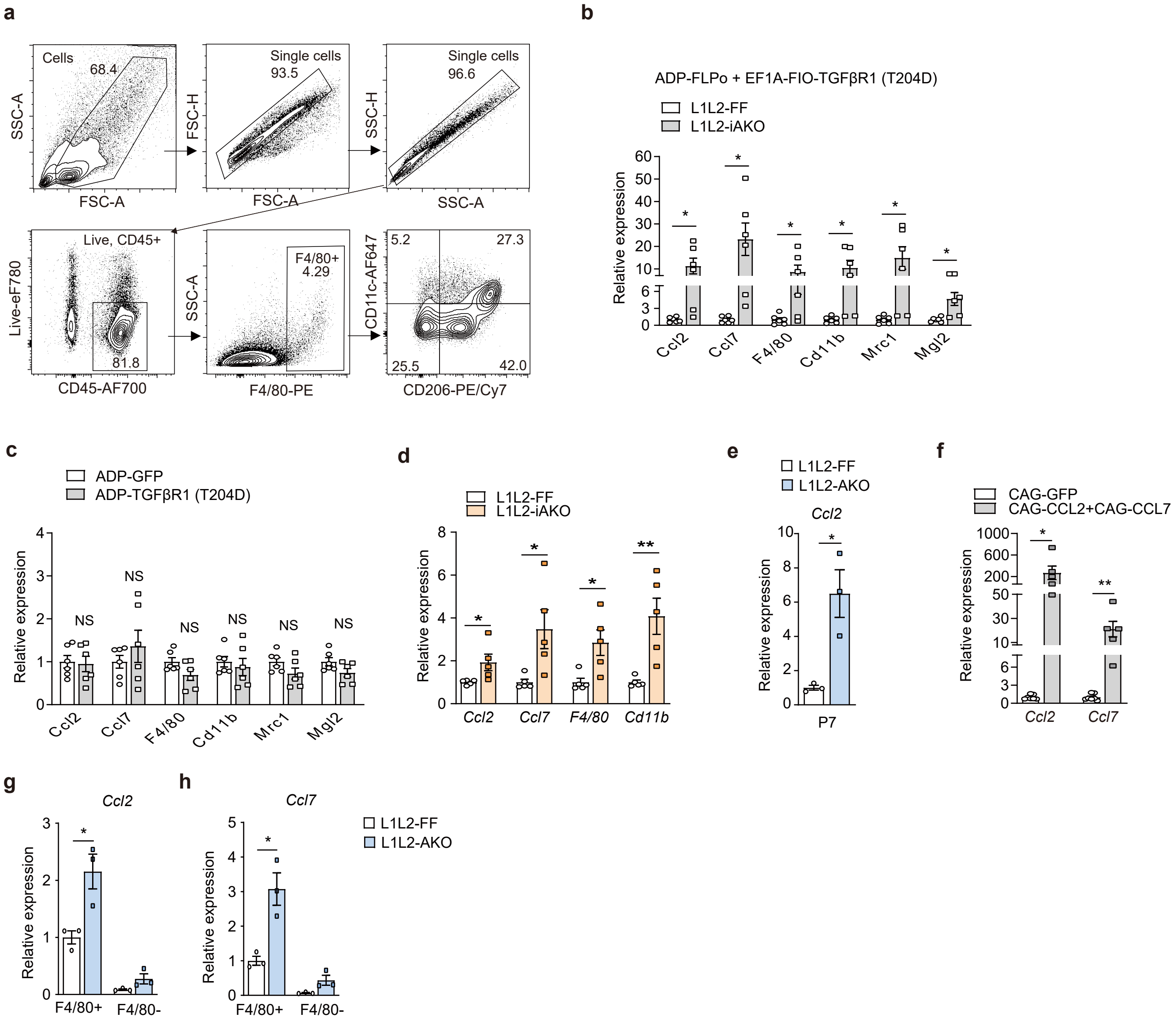

Supplementary Figure 8. *Lats1/2* deletion induces inflammatory responses in scWAT.

a, Gating strategy of flow cytometry for macrophages. **b**, L1L2-FF or L1L2-iAKO mice that were locally injected with AAV-ADP-FLPo and AAV-EF1A-FIO-TGFβR1 (T204D). mRNA expression of inflammatory genes in scWAT (n=6 mice). **c**, L1L2-FF mice that were locally injected with AAV-ADP-GFP or AAV-ADP-TGFβR1 (T204D). mRNA expression of inflammatory genes in scWAT (n=6 mice). **d**, mRNA expression of inflammatory markers in L1L2-FF or L1L2-iAKO mice (n=5). **e**, mRNA expression of *Ccl2* in adipocytes of pooled P7 male L1L2-FF or L1L2-AKO scWAT (n=3). One dot represents a mean value of biologically independent samples from four male mice. **f**, Overexpression efficiency of AAV-CAG-GFP (n=7 mice), AAV-CAG-CCL2 and AAV-CAG-CCL7 (n=5 mice) in scWAT. **g**, **h**, F4/80$^-$ and F4/80$^+$ cells were magnetically sorted from pooled L1L2-FF or L1L2-AKO scWAT SVF and analyzed for mRNA expression of (**g**) *Ccl2* or (**h**) *Ccl7* (n=3). One dot represents a mean value of biologically independent samples from three male mice.. Data are means ± SEM. Two-tailed unpaired student's *t*-test; $*P < 0.05$, $**P < 0.01$, $***P < 0.001$. Exact *P* values are provided in a Source Data file.

Supplementary Figure 9

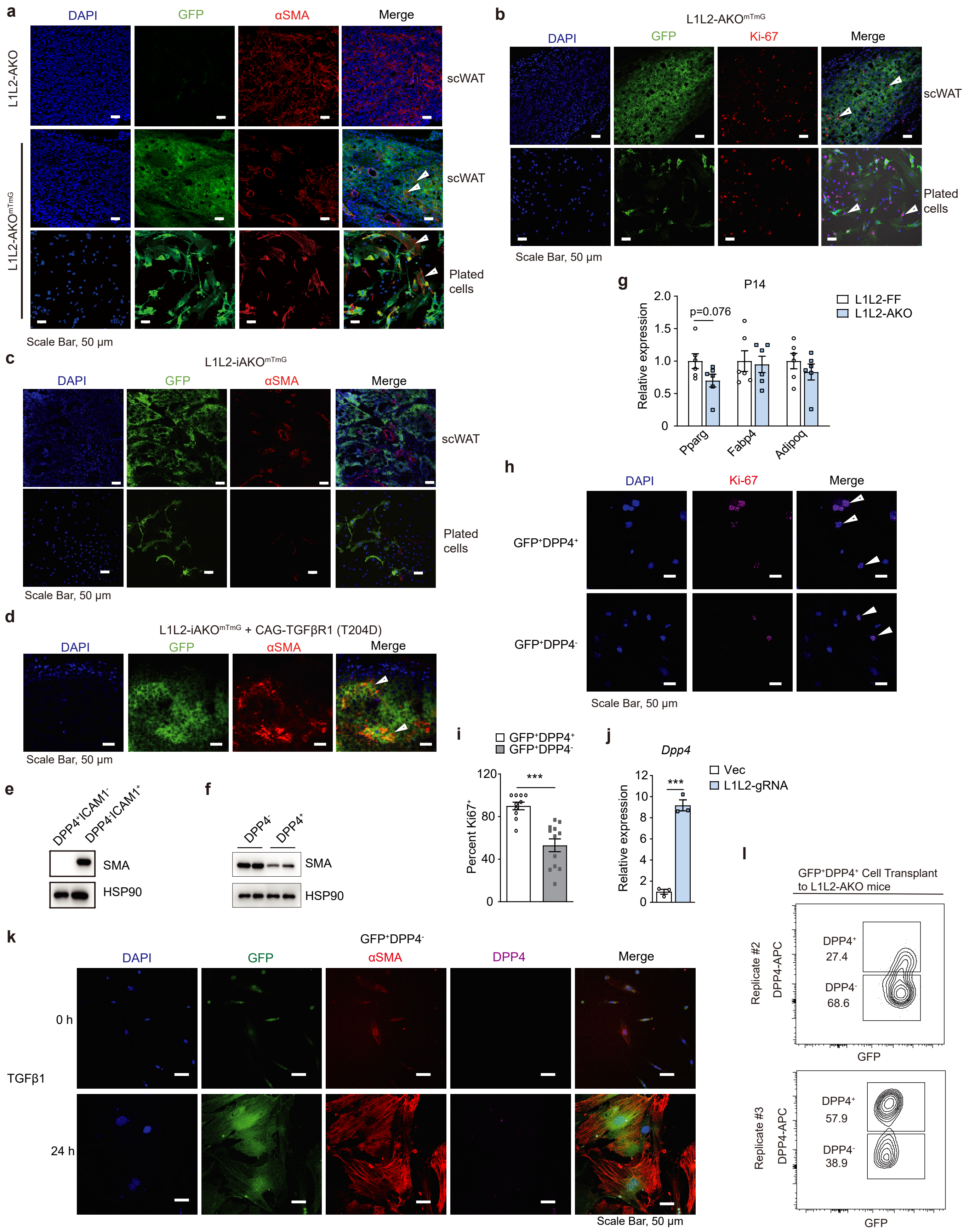

Supplementary Figure 9. *Lats1/2* deletion promotes the cell fate conversion from adipocyte to myofibroblast.

a, Representative sections of scWAT of P28 L1L2-AKO^{mTmG} mice stained for αSMA (AF647, red), nucleus (DAPI, blue) and membrane GFP (green). Arrowheads show examples of GFP^{+}αSMA^{+} cells. Independent experiments were performed three times with similar results. **B**, Representative sections of scWAT of P28 L1L2-AKO^{mTmG} mice stained for Ki-67 (eFluor 660, red), nucleus (DAPI, blue). Arrowheads show examples of GFP^{+}Ki-67^{+} cells. Independent experiments were performed twice with similar results. **C**, Representative sections of scWAT of L1L2-iAKO^{mTmG} mice stained for αSMA (AF647, red), nucleus (DAPI, blue) and membrane GFP (green). Independent experiments were performed twice with similar results. **D**, Representative sections of scWAT of L1L2-iAKO^{mTmG} transduced with AAV-CAG- TGFβR1 (T204D) and stained for αSMA (AF647, red), nucleus (DAPI, blue) and membrane GFP (green). Arrowheads indicate GFP^{+}αSMA^{+} cells. Independent experiments were performed twice with similar results. **e**, Immunoblot analysis of αSMA protein expression in sorted DPP4^{+} or DPP4^{-} cells from WT scWAT SVF. Independent experiments were performed twice with similar results. **f**, Immunoblot analysis of αSMA protein expression in sorted DPP4^{+}ICAM1^{-} or DPP4^{-}ICAM^{+} cells from WT scWAT SVF. Independent experiments were performed twice with similar results. **g**, mRNA expression of adipocyte markers of P14 L1L2-FF or L1L2-AKO scWAT (n=6 mice). **h**, GFP^{+}DPP4^{+} or GFP^{+}DPP4^{-} cells sorted from L1L2-AKO^{mTmG} scWAT SVF were plated and stained for Ki-67 (eFluor 660, purple) and nucleus (DAPI, blue). Independent experiments were performed twice with similar results. **i**, Quantification of Ki-67^{+} cell percentages of GFP^{+}DPP4^{+} (n=10 visual fields) or GFP^{+}DPP4^{-} (n=12 visual fields) cells. **j**, RT-qPCR analysis of *Dpp4* mRNA expression in differentiated

adipocytes (from *Cas9*$^{Tg/+}$ scWAT SVF) transduced with Vec or L1L2-gRNA (n=3 biologically independent cell cultures). **k**, Sorted GFP^{+}DPP4^{-} cells from P21 L1L2-AKO$^{LSL\text{-}CAS9\text{-}EGFP}$ scWAT SVF were treated with TGF-β1 (10 ng/ml) for 0 h or 24 h. Cells were stained with αSMA (red), DPP4 (purple), nucleus (DAPI, blue) and GFP (green). Independent experiments were performed twice with similar results. **l**, The other two recipient mice as in Fig. 5g. Donor cells were from scWAT SVF of L1L2-AKO^{mTmG} (upper) or L1L2-AKO$^{LSL\text{-}CAS9\text{-}EGFP}$ (lower) mice. Data are means ± SEM. Two-tailed unpaired student's *t*-test; *$P < 0.05$, **$P < 0.01$, ***$P < 0.001$. Exact *P* values are provided in a Source Data file.

Supplementary Figure 10

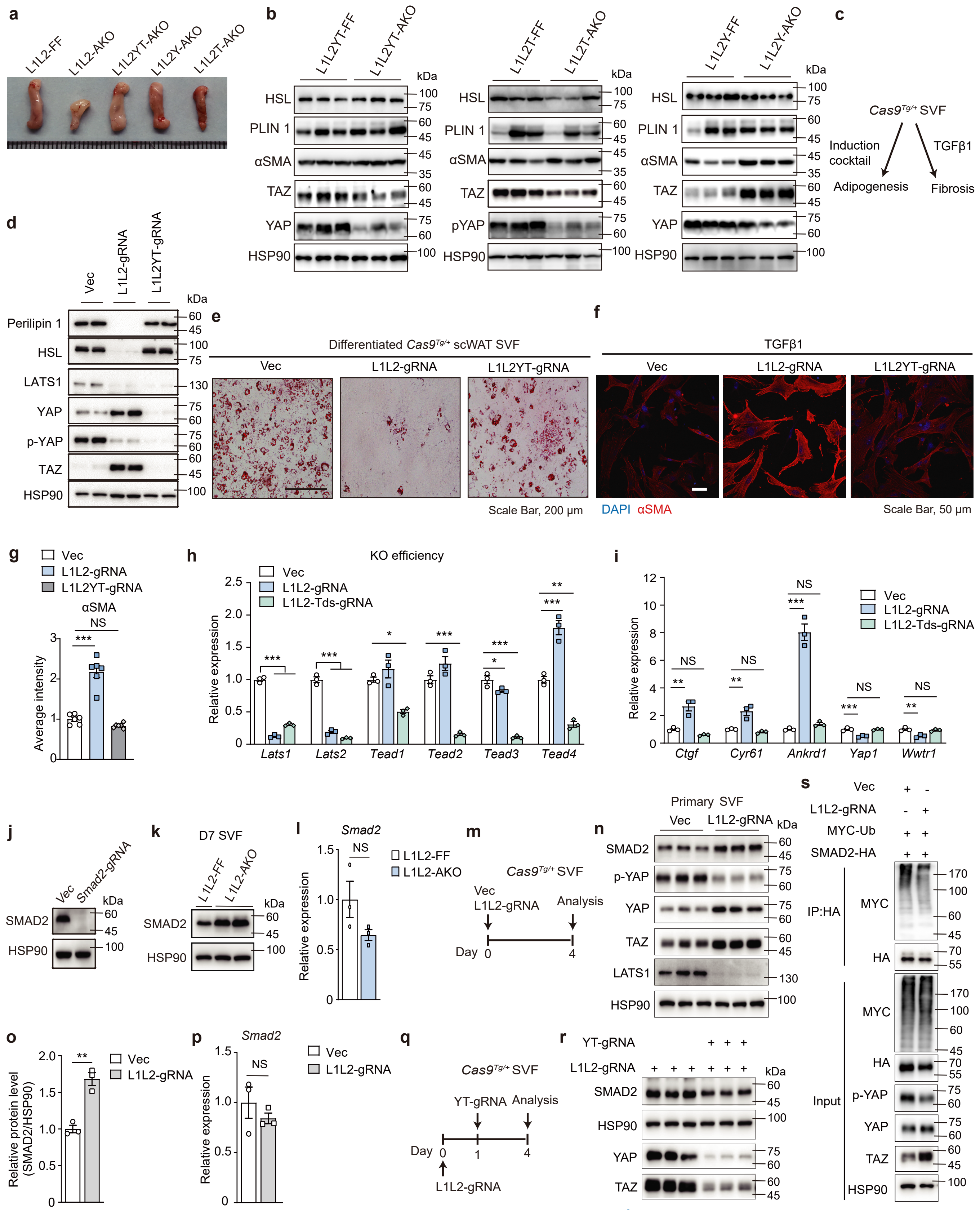

Supplementary Figure 10. The LATS1/2-YAP/TAZ-TEADs axis regulates adipocyte identity.

a, Representative images of scWAT of the indicated mouse strains. **b**, Immunoblot analysis of HSL, Perilipin 1 and αSMA protein expression in scWAT of the indicated mouse strains (n=3 mice). **c-g**, Primary $Cas9^{Tg/+}$ SVF treated with TGFβ1 or adipogenic cocktail. **c**, Experimental scheme. **d**, Immunoblot analysis of adipocyte markers in differentiated $Cas9^{Tg/+}$ SVF. **e**, Oil red staining of differentiated $Cas9^{Tg/+}$ SVF transduced with Vec, L1L2-gRNA or L1L2YT-gRNA. Independent experiments were performed twice with similar results. **f**, Immunostaining of αSMA^{+} primary SVF cells treated with TGFβ1 (10 ng/mL) for 24 h. Independent experiments were performed twice with similar results. **g**, Quantification of average intensity of αSMA in (**f**) (n=6 visual fields). **h**, **i**, RT-qPCR analysis of the knockdown efficiency of (**h**) *Lats1/2* and *Tead1/2/3/4* and (**i**) the Hippo pathway downstream gene expression in differentiated adipocytes (n=3 biologically independent cell cultures). **j**, Immunoblot analysis of the specificity of SMAD2 antibody. Independent experiments were performed twice with similar results. **k**, Immunoblot analysis of SMAD2 protein expression in differentiated adipocytes from SVF of L1L2-FF or L1L2-AKO scWAT. Independent experiments were performed twice with similar results. **l**, mRNA expression of *Smad2* in differentiated adipocytes from SVF of L1L2-FF or L1L2-AKO scWAT (n=3 biologically independent cell cultures). **m-p**, Primary $Cas9^{Tg/+}$ SVF transduced with Vec or L1L2-gRNA for 4 days. **m**, Experimental timeline. **n**, SMAD2 protein expression. **o**, Quantification of SMAD2 protein expression (n=3 biologically independent cell cultures). **p**, mRNA expression of *Smad2* (n=3 biologically independent cell cultures). **q**, **r**, Primary $Cas9^{Tg/+}$ SVF transduced with L1L2-gRNA on day 0, followed by YT-gRNA on day 1. **q**, Experimental timeline. **r**, Immunoblot

analysis of SMAD2 protein expression. Independent experiments were performed twice with similar results. **s**, Differentiated adipocytes were transduced with Vec, L1L2-gRNA1, MYC-Ub and SMAD2-HA. Immunoprecipitation (IP) followed by western blot assay was performed. Independent experiments were performed twice with similar results. Data are means ± SEM. One-way ANOVA with Bonferroni's multiple-comparisons test in (**g**), (**h**) and (**i**); Two-tailed unpaired student's *t*-test in (**l**), (**o**) and (**p**); $*P < 0.05$, $**P < 0.01$, $***P < 0.001$; NS, not significant. Exact *P* values are provided in a Source Data file.

Supplementary Figure 11

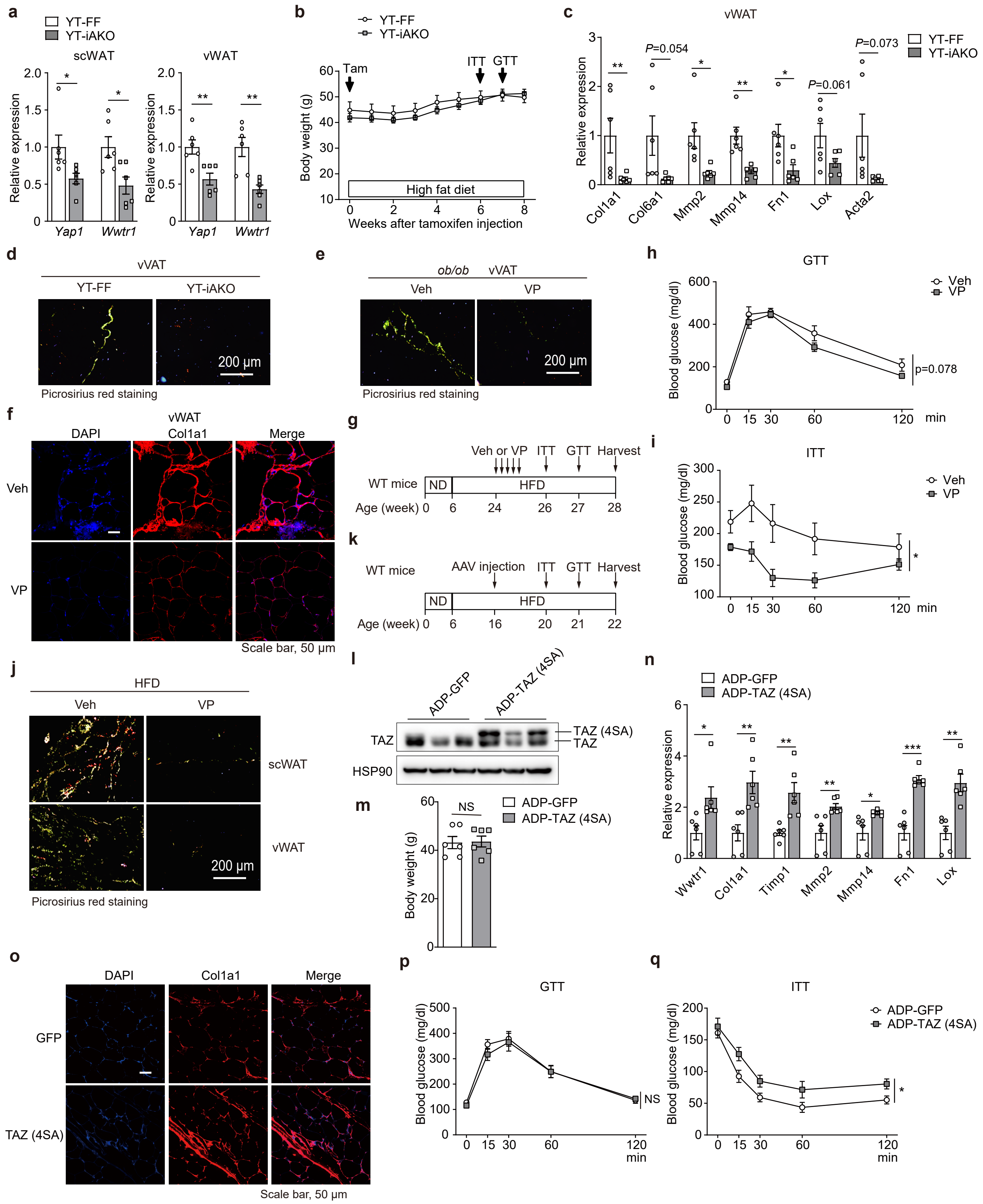

Supplementary Figure 11. Targeting the Hippo pathway in precursor cells and adipocytes to treat obesity-induced AT fibrosis and metabolic dysfunction. **a-d**, YT-FF and YT-iAKO mice were fed a HFD for 19 weeks. **a**, Knockout efficiency of *Yap1* or *Wwtr1* in scWAT or vVAT (n=6 mice). **b**, Body weight of YT-FF and YT-iAKO mice under a high-fat diet before and after 5 doses of tamoxifen (Tam) (50 mg/kg) treatment (n=6 mice). **c**, mRNA expression of fibrosis markers in vWAT (n=6 mice). **d**, Representative vWAT sections with Picrosirius red staining. **e**, Representative vWAT sections with Picrosirius red staining of 12-week-old *ob/ob* mice administrated with Veh or VP. Independent experiments were performed three times with similar results. **f,** Representative sections of vVAT stained for Col1a1 (red) and nucleus (DAPI, blue) of *ob/ob* mice that were i.p. injected with Veh or VP. Independent experiments were performed twice with similar results. **g-j**, Mice fed a HFD for 18 weeks were i.p. injected with Veh or VP (25 mg/kg) for 5 doses every other day. **g**, Experimental scheme. **h**, GTT (n=6 mice). **i**, ITT (n=6 mice). **j**, Representative scWAT sections with Picrosirius red staining in scWAT and vVAT. Independent experiments were performed three times with similar results. **k-q**, WT mice were fed a HFD for 10 weeks followed by an injection with AAV-ADP-GFP, AAV-ADP-TAZ (4SA) in scWAT for 4 weeks. **k**, Experimental scheme. **l**, Immunoblot analysis of protein expression of TAZ(4SA) in scWAT. **m**, Body weight (n=6 mice). **n**, mRNA expression of fibrosis markers (n=6). **o**, Representative scWAT sections with IF staining of Col1a1. **p**, GTT (n=6 mice). **q**, ITT (n=6 mice). Data are means ± SEM. Two-tailed unpaired student's *t*-test in (**a**), (**b**), (**c**), (**m**), (**n**); Two-way ANOVA with Bonferroni's multiple-comparisons test in (**h**), (**i**), (**p**), (**q**); $^{*}P < 0.05$, $^{**}P < 0.01$, $^{***}P < 0.001$. Exact *P* values are provided in a Source Data file.

Supplementary Table 1

Sequences of qRT-PCR primers used in this study.

Gene	Forward primer	Reverse primer	Note
Fn1	GATGTCCGAACAGCTATTTACCA	CCTTGCGACTTCAGCCACT	
Mmp2	GGACAAGTGGTCCGCGTAAA	CCGACCGTTGAACAGGAAGG	
Mmp14	CAGTATGGCTACCTACCTCCAG	GCCTTGCCTGTCACTTGTAAA	
Lox	CAGCCACATAGATCGCATGGT	GCCGTATCCAGGTCGGTTC	
Acta2	GTCCCAGACATCAGGGAGTAA	TCGGATACTTCAGCGTCAGGA	
Tagln	CAACAAGGGTCCATCCTACGG	ATCTGGGCGGCCTACATCA	
Col1a1	TAAGGGTCCCCAATGGTGAGA	GGGTCCCTCGACTCCTACAT	
Col3a1	CTGTAACATGGAAACTGGGGAAA	CCATAGCTGAACTGAAAACCAC C	
Col6a1	CCGACTGCGCCATTAAGAAG	CCCCGCATGGTTCCTTGTAG	
Tgfb1	ATTCCTGGCGTTACCTTGG	AGCCCTGTATTCCGTCTCCT	
Tgfb2	TCGACATGGATCAGTTTATGCG	CCCTGGTACTGTTGTAGATGGA	
Tgfb3	CCTGGCCCTGCTGAACTTG	TTGATGTGGCCGAAGTCCAAC	
Tgfbr1	CAGCTCCTCATCGTGTTGGTG	GCACATACAAATGGCCTGTCTC	
Tgfbr2	GATGTCATGGCCAGCGACAA	TGTTCTTGTCGTTCTTCCTC	
Smad2	ATGTCGTCCATCTTGCCATTC	AACCGTCCTGTTTTCTTTAGCTT	
Timp1	CGAGACCACCTTATACCAGCG	ATGACTGGGGTGTAGGCGTA	
Ccl2	TTAAAAACCTGGATCGGAACCAA	GCATTAGCTTCAGATTTACGGGT	
Ccl7	CCACATGCTGCTATGTCAAGA	ACACCGACTACTGGTGATCCT	
Mrc1 (CD206)	CTCTGTTCAGCTATTGGACGC	CGGAATTTCTGGGATTCAGCTTC	
Mgl1 (CD301)	TGAGAAAGGCTTTAAGAACTGGG	GACCACCTGTAGTGATGTGGG	
Nos2	GAGCAACTACTGCTGGTGGT	TCAGAGTCTGCCCATTGCTG	
F4/80	TGACTCACCTTGTGGTCCTAA	CTTCCCAGAATCCAGTCTTTCC	
CD11b	CCTTCATCAACACAACCAGAGTGG	CGAGGTGCTCCTAAAACCAAGC	
Pparg	CAAGAATACCAAAGTGCGATCAA	GAGCTGGGTCTTTTCAGAATAAT AAG	
Fabp4	ACACCGAGATTTCCTTCAAACTG	CCATCTAGGGTTATGATGCTCTT C	
Adipoq	GCACTGGCAAGTTCTACTGCAA	GTAGGTGAAGAGAACGGCCTTG T	
Glut4	GTGACTGGAACACTGGTCCTA	CCAGCCACGTTGCATTGTAG	
Hsl	CCAGCCTGAGGGCTTACTG	CTCCATTGACTGTGACATCTCG	
Lats1	ATCTCCCGAATCTCCCCTGTT	GTCGCCCTGGTGTAAAGTTAAA	
Lats1	GAAAGACGTTCTGCTCCGAA	ATCGTGCCAGATTTTCAGGA	for detection of KO efficiency of L1-gRNA1
Lats1	GGAGTTCAGAATGGTGGTGG	GGAACGTTTCCATTGGCGAA	for detection of KO efficiency of L1-gRNA2
Lats2	GGCTTTATCCACCGGGACAT	AATCCAGTGCAGAGGCCAAA	
Lats2	TGGAGCAGGAAATGGCCAAA	CGTGAGTGTCCAGCTTACAA	for detection of KO efficiency of L2-gRNA1
Lats2	GCAGACGGGCAGTAGGAGTA	CCTCGTAGTTTGCACCACCC	for detection of KO efficiency of L2-gRNA2

Yap1	TGAGATCCCTGATGATGTACCAC	TGTTGTTGTCTGATCGTTGTGAT	
Wwtr1	CCGGTTCCGGGGATAAAGAT	GTTGAAGAGGGCTTCGAGGT	
36B4	TCACTGTGCCAGCTCAGAAC	ATCAGCTGCACATCACTCAGA	
Tead1	AAGCCGATTGACAACGACGC	CCTTGTCTTTCCCGTCCTGA	
Tead2	ATGGGGGATCCCCGGACTGG	ATGATCTTGCGACGGCCACA	
Tead3	AAGGGTCTGGACAACGATGC	CTGTTTTCTTGTCCTGGTTT	
Tead4	TGGAGCTCTCCCGACTCCCC	TGCGATCAGCTCATTCCGAC	
Ctgf	GGACACCTAAAATCGCCAAGC	ACTTAGCCCTGTATGTCTTCACA	
Cyr61	TAAGGTCTGCGCTAAACAACTC	CAGATCCCTTTCAGAGCGGT	
Ankrd1	TGCGATGAGTATAAACGGACG	GTGGATTCAAGCATATCTCGGA A	

Supplementary Table 2

Sequences of gRNAs used in this study.

gRNA	Sequence	Note
Lats1-gRNA1	AGACGTTCTGCTCCGAAATC	[68]
Lats1-gRNA2	ACGTTTCCATTGGCGAATGA	
Lats2-gRNA1	GAGTGTCCAGCTTACAAGCG	[68]
Lats2-gRNA2	GCTGGGTGGTGCAAACTACG	
Yap-gRNA	GATCAGACAACAACATGGC	
Taz-gRNA	TCACGTCATAGGACTGCTGG	
Tead1-gRNA	CCGATTGACAACGACGCGGA	
Tead2-gRNA	ATCTTGCGACGGCCACAGGG	
Tead3-gRNA	GTCTGGACAACGATGCGGA	
Tead4-gRNA	AGCTCTCCCGACTCCCCCGA	
Smad2-gRNA	CTTGCCATTCACTCCGCCAG	
