# Supplementary material for: The Hippo pathway links adipocyte plasticity to adipose tissue fibrosis
Source: Nat Commun. 2022 Oct 13;13:6030. doi: 10.1038/s41467-022-33800-0 (PMC9562301; doi:10.1038/s41467-022-33800-0)
Supplement: Supplementary file 2 — Reporting Summary [file 41467_2022_33800_MOESM2_ESM.pdf]

nature portfolio

Corresponding author(s): Yifu Qiu

Last updated by author(s): Aug 14, 2022

Reporting Summary

Nature Portfolio wishes to improve the reproducibility of the work that we publish. This form provides structure for consistency and transparency in reporting. For further information on Nature Portfolio policies, see our Editorial Policies and the Editorial Policy Checklist.

Statistics

For all statistical analyses, confirm that the following items are present in the figure legend, table legend, main text, or Methods section.

n/a	Confirmed	
☐	☒	The exact sample size (*n*) for each experimental group/condition, given as a discrete number and unit of measurement
☐	☒	A statement on whether measurements were taken from distinct samples or whether the same sample was measured repeatedly
☐	☒	The statistical test(s) used AND whether they are one- or two-sided<br>*Only common tests should be described solely by name; describe more complex techniques in the Methods section.*
☒	☐	A description of all covariates tested
☐	☒	A description of any assumptions or corrections, such as tests of normality and adjustment for multiple comparisons
☐	☒	A full description of the statistical parameters including central tendency (e.g. means) or other basic estimates (e.g. regression coefficient) AND variation (e.g. standard deviation) or associated estimates of uncertainty (e.g. confidence intervals)
☐	☒	For null hypothesis testing, the test statistic (e.g. *F*, *t*, *r*) with confidence intervals, effect sizes, degrees of freedom and *P* value noted<br>*Give P values as exact values whenever suitable.*
☒	☐	For Bayesian analysis, information on the choice of priors and Markov chain Monte Carlo settings
☒	☐	For hierarchical and complex designs, identification of the appropriate level for tests and full reporting of outcomes
☒	☐	Estimates of effect sizes (e.g. Cohen's *d*, Pearson's *r*), indicating how they were calculated

Our web collection on statistics for biologists contains articles on many of the points above.

Software and code

Policy information about availability of computer code

Data collection	Applied Biosystems StepOnePlus Real-Time PCR Systems, Tanon 5200 Chemiluminescent Imaging System, CytoFLEX S flow cytometer, Olympus BX51 Microscopy, Olympus BX53 Microscopy, BioTek Multi-Detection Microplate Reader, EVOS® FL Imaging Systems, GE Lunar PIXImus densitometer, Carl ZEISS LSM 710 NLO Microscopy, Nikon AIR-si Microscopy, BD FACSAria III cytometer, BGISEQ-500 sequencer.
Data analysis	ImageJ v10 was used for immunoblot densitometry analysis and immunofluorescence analysis, GraphPad Prism v8.0.2, FastQC v0.11.9, SAMtools v1.3.1, StringTie v1.3.5, HISAT2 v2.1.0, Flowjo v10, Microsoft Excel 2019, R v3.6.3, CytExpert v2.3.0.84

For manuscripts utilizing custom algorithms or software that are central to the research but not yet described in published literature, software must be made available to editors and reviewers. We strongly encourage code deposition in a community repository (e.g. GitHub). See the Nature Portfolio guidelines for submitting code & software for further information.

Data

Policy information about availability of data

All manuscripts must include a data availability statement. This statement should provide the following information, where applicable:

- Accession codes, unique identifiers, or web links for publicly available datasets
- A description of any restrictions on data availability
- For clinical datasets or third party data, please ensure that the statement adheres to our policy

Our RNA-seq data generated in this study have been deposited in the NCBI Gene Expression Omnibus repository under accession code GSE184826 [https://www.ncbi.nlm.nih.gov/geo/query/acc.cgi?acc=GSE184826]. The RNA-seq data used in this study are publicly available in the NCBI Gene Expression Omnibus repository under the accession numbers GSE141432 [https://www.ncbi.nlm.nih.gov/geo/query/acc.cgi?acc=GSE141432], GSE152991[https://www.ncbi.nlm.nih.gov/geo/query/acc.cgi?acc=GSE152991]. Gene list involved in Hippo pathway was from Hallmark gene sets from molecular signatures database

March 2021

v7.5.1 (https://www.gsea-msigdb.org/gsea/msigdb/index.jsp). Source data are provided with this paper.

Field-specific reporting

Please select the one below that is the best fit for your research. If you are not sure, read the appropriate sections before making your selection.

- [x] Life sciences
- [ ] Behavioural & social sciences
- [ ] Ecological, evolutionary & environmental sciences

For a reference copy of the document with all sections, see nature.com/documents/nr-reporting-summary-flat.pdf

Life sciences study design

All studies must disclose on these points even when the disclosure is negative.

Sample size	Sample size was choosen based on previous experiments (doi: 10.1016/j.cell.2014.03.066; doi: 10.1038/ni.3709; doi: 10.1038/ni.3728) or based on pilot experiments to ensure the possibility of statistical analysis and to minimize the use of experimental animals based on the 3R principles.
Data exclusions	No data was excluded.
Replication	All the experiments and data shown in this work have been repeated independently at least twice with similar results as indicated in the legends.
Randomization	Ob/ob and HFD-induced obese mice were randomly allocated to groups for verteporfin (VP) treatment. Randomization was not done to other mice that were grouped based on genotypes.
Blinding	Blinding was not done as most of the experiments were performed by a single person and the data was acquired and analyzed by the same person. It was not feasible during the course of the study to have at least two individuals for each experiment. No data was excluded in this study and all analyses were performed quantitatively and not subjectively.

Reporting for specific materials, systems and methods

We require information from authors about some types of materials, experimental systems and methods used in many studies. Here, indicate whether each material, system or method listed is relevant to your study. If you are not sure if a list item applies to your research, read the appropriate section before selecting a response.

Materials & experimental systems

n/a	Involved in the study	
[ ]	[x]	Antibodies
[ ]	[x]	Eukaryotic cell lines
[x]	[ ]	Palaeontology and archaeology
[ ]	[x]	Animals and other organisms
[x]	[ ]	Human research participants
[x]	[ ]	Clinical data
[x]	[ ]	Dual use research of concern

Methods

n/a	Involved in the study	
[x]	[ ]	ChIP-seq
[ ]	[x]	Flow cytometry
[x]	[ ]	MRI-based neuroimaging

Antibodies

Antibodies used

Antibodies for immunoblot
Primary Antibodies:
Rabbit anti-αSMA Cell Signaling #19245 1:5000
Mouse anti-HSP90α/β Santa Cruz Biotech. #sc-13119 clone:F-8 1:10000
Mouse anti-α-Tubulin Sigma-Aldrich #T6199 1:10000
Mouse anti-Lamin B1 Protein tech #66095-1-Ig 1:10000
Rabbit anti-YAP Cell Signaling #14074 1:1000
Rabbit anti-YAP Cell Signaling #4912 1:1000
Rabbit anti-TAZ Cell Signaling #83669 1:1000
Rabbit anti-TAZ Cell Signaling #4883 1:1000
Rabbit anti-LATS1 Cell Signaling #3477 1:1000
Rabbit anti-LATS2 Bethyl Laboratories#A300-479A 1:1000
Rabbit p-YAP (Ser 112) Cell Signaling #4911 1:1000
Rabbit p-TAZ (Ser 89) Cell Signaling #59971 1:1000
Rabbit anti-SMAD2/3 Cell Signaling #8685 1:1000
Rabbit anti-p-SMAD2 (Ser465/467)/SMAD3 (Ser423/425) Cell Signaling #8828 1:1000

Mouse anti-GFP Thermo Scientific #A-11120 1:1000
Rabbit anti-HSL Cell Signaling #4107 1:1000
Mouse anti-Perilipin 1 Vala Sciences #4854 1:10000
Mouse anti-Flag Abmart #M20008 1:5000
Rabbit anti-HA Cell Signaling #3724 1:1000
Rabbit anti-Myc Cell Signaling #2276 1:2000
Caspase 3 (Cell Signaling, 9662) 1:1000
MST1 (Cell Signaling, 3682) 1:1000
MST2 (Cell Signaling, 3952) 1:1000
p-SAPK/JNK (Thr183/Tyr185) (Cell Signaling, 4668) 1:1000
SAPK/JNK (Cell Signaling, 9252) 1:1000
p-AKT (Ser473) (Cell Signaling, 4060) 1:1000
AKT (Cell Signaling, 9272) 1:1000
p-p38 MAPK (Thr180/Tyr182) (Cell Signaling, 4511) 1:1000
p38 MAPK (Cell Signaling, 8690) 1:1000
p-MLC2 (Ser19) (Cell Signaling, 3671) 1:1000
MLC2 (Proteintech, 10906-1-AP) 1:1000
ERK1/ERK2 (ABclonal, A10613) 1:1000
p-ERK1(T202/Y204)/ERK2(T185/Y187) (ABclonal, AP0472) 1:1000
Col1a1 (Cell Signaling, 72026) 1:1000

Secondary antibodies:
Goat anti-mouse IgG:HRP Thermo Scientific #32430 1:10000
Goat anti-rabbit IgG:HRP Thermo Scientific #31460 1:10000

Antibodies for flow cytometry
CD16/32 BioLegend #101302, clone: 93, 1:1000
CD45-PerCP/Cy5.5 BioLegend #103131 clone: 30-F11 1:1000
CD45-AF700 BD Biosciences #560510 clone: 30-F11 1:800
CD31-PerCP/Cy5.5 BioLegend #102419 clone:390 1:300
F4/80-PE Cell Signaling #64763 1:80
CD11c-AF647 BioLegend #117314 clone: N418 1:100
CD206-PE Cy7 BioLegend #141720 clone: C068C2 1:100
CD26 (DPP4)-APC BioLegend #137807 clone: H194-112 1:100
CD54 (ICAM1)-PE/Cy7 BioLegend #116121 clone: YN1/1.7.4 1:100
Anti-F4/80-Biotin Miltenyi Biotech. #130-116-514 1:100

Antibodies for immunohistochemistry
Rabbit anti-F4/80 #70076 Cell Signaling 1:200
Goat anti-rabbit HRP-conjugated secondary antibody ZSGB-Bio #ZB-2010 1:200
Biotin-conjugated goat anti-rabbit IgG (H+L) #ZB-2010 ZSGB-Bio 1:200

Antibodies for immunofluorescence
Rabbit anti-αSMA Cell Signaling #19245 1:5000
Rabbit anti-CD26 (DPP4)-APC BioLegend #137807 clone: H194-112 1:100
Rabbit anti-Ki67-eFluor 660 eBioscience #50-5698-82 1:200
Goat anti-Rabbit-IgG: Alexa Fluor 647 Thermo Scientific #A21244 1:300
Goat anti-Rabbit-IgG: PE Thermo Scientific #12-4739-81 1:400
Rabbit anti-p-SMAD2 (S465/S467) Cell Signaling #18338, 1:100
Rabbit anti-Col1a1 Cell Signaling #72026 1:100

Validation

All antibodies used were purchased from commercial vendors. All the primary antibodies used in this study were validated in mice.
Antibodies for immunoblot
Primary Antibodies:
Rabbit anti-αSMA Cell Signaling #19245 1:5000
https://www.cellsignal.cn/datasheet.jsp?productId=19245&images=1&size=A4
Mouse anti-HSP90α/β Santa Cruz Biotech. #sc-13119 clone:F-8 1:10000
https://www.scbt.com/p/hsp-90alpha-beta-antibody-f-8?requestFrom=search
Mouse anti-α-Tubulin Sigma-Aldrich #T6199 1:10000
https://www.sigmaaldrich.cn/CN/zh/product/sigma/t6199
Mouse anti-Lamin B1 Protein tech #66095-1-Ig 1:10000
https://www.ptgcn.com/products/LMNB1-Antibody-66095-1-Ig.htm
Rabbit anti-YAP Cell Signaling #14074 1:1000
https://www.cellsignal.cn/datasheet.jsp?productId=14074&images=1&size=A4
Rabbit anti-YAP Cell Signaling #4912 1:1000
https://www.cellsignal.cn/datasheet.jsp?productId=4912&images=1&size=A4
Rabbit anti-TAZ Cell Signaling #83669 1:1000
https://www.cellsignal.cn/datasheet.jsp?productId=83669&images=1&size=A4
Rabbit anti-TAZ Cell Signaling #4883 1:1000

https://www.cellsignal.cn/datasheet.jsp?productId=4883&images=1&size=A4
Rabbit anti-LATS1 Cell Signaling #3477 1:1000
https://www.cellsignal.cn/datasheet.jsp?productId=3477&images=1&size=A4
Rabbit anti-LATS2 Bethyl Laboratories#A300-479A 1:1000
doi: 10.1016/j.cell.2016.11.005.
Rabbit p-YAP (Ser 112) Cell Signaling #4911 1:1000
https://www.cellsignal.cn/datasheet.jsp?productId=4911&images=1&size=A4
Rabbit p-TAZ (Ser 89) Cell Signaling #59971 1:1000
https://www.cellsignal.cn/products/primary-antibodies/phospho-taz-ser89-e1x9c-rabbit-mab/59971?site-search-type=Products&N=4294956287&Ntt=59971&fromPage=plp&_requestid=4064841
Rabbit anti-SMAD2/3 Cell Signaling #8685 1:1000
https://www.cellsignal.cn/datasheet.jsp?productId=8685&images=1&size=A4
Rabbit anti-p-SMAD2 (Ser465/467)/SMAD3 (Ser423/425) Cell Signaling #8828 1:1000
https://www.cellsignal.cn/datasheet.jsp?productId=8828&images=1&size=A4
Mouse anti-GFP Thermo Scientific #A-11120 1:1000
https://www.thermofisher.cn/cn/zh/antibody/product/GFP-Antibody-clone-3E6-Monoclonal/A-11120
Rabbit anti-HSL Cell Signaling #4107 1:1000
https://www.cellsignal.cn/datasheet.jsp?productId=4107&images=1&size=A4
Mouse anti-Perilipin 1 Vala Sciences #4854 1:10000
https://valasciences.com/reagent-4854/
Mouse anti-Flag Abmart #M20008 1:5000
http://www.ab-mart.com.cn/page.aspx?node=%2060%20&id=%20968
Rabbit anti-HA Cell Signaling #3724 1:1000
https://www.cellsignal.cn/datasheet.jsp?productId=3724&images=1&size=A4
Rabbit anti-Myc Cell Signaling #2276 1:2000
https://www.cellsignal.cn/datasheet.jsp?productId=2276&images=1&size=A4
Rabbit anti-Caspase 3 (Cell Signaling, 9662) 1:1000
https://www.cellsignal.cn/datasheet.jsp?productId=9662&images=1&size=A4
Rabbit anti-MST1 (Cell Signaling, 3682) 1:1000
https://www.cellsignal.cn/datasheet.jsp?productId=3682&images=1&size=A4
Rabbit anti-MST2 (Cell Signaling, 3952) 1:1000
https://www.cellsignal.cn/datasheet.jsp?productId=3952&images=1&size=A4
Rabbit anti-p-SAPK/JNK (Thr183/Tyr185) (Cell Signaling, 4668) 1:1000
https://www.cellsignal.cn/datasheet.jsp?productId=4668&images=1&size=A4
Rabbit anti-SAPK/JNK (Cell Signaling, 9252) 1:1000
https://www.cellsignal.cn/datasheet.jsp?productId=9252&images=1&size=A4
Rabbit anti-p-AKT (Ser473) (Cell Signaling, 4060) 1:1000
https://www.cellsignal.cn/datasheet.jsp?productId=4060&images=1&size=A4
Rabbit anti-AKT (Cell Signaling, 9272) 1:1000
https://www.cellsignal.cn/datasheet.jsp?productId=9272&images=1&size=A4
Rabbit anti-p-p38 MAPK (Thr180/Tyr182) (Cell Signaling, 4511) 1:1000
https://www.cellsignal.cn/datasheet.jsp?productId=4511&images=1&size=A4
Rabbit anti-p38 MAPK (Cell Signaling, 8690) 1:1000
https://www.cellsignal.cn/datasheet.jsp?productId=8690&images=1&size=A4
Rabbit anti-p-MLC2 (Ser19) (Cell Signaling, 3671) 1:1000
https://www.cellsignal.cn/datasheet.jsp?productId=3671&images=1&size=A4
Rabbit anti-MLC2 (Proteintech, 10906-1-AP) 1:1000
https://www.ptgcn.com/products/MYL2-Antibody-10906-1-AP.htm
Mouse anti-ERK1/ERK2 (ABclonal, A10613) 1:1000
https://abclonal.com.cn/catalog/A10613
p-ERK1(T202/Y204)/ERK2(T185/Y187) (ABclonal, AP0472) 1:1000
https://abclonal.com.cn/catalog/AP0472
Rabbit anti-Col1a1 (Cell Signaling, 72026) 1:1000
https://www.cellsignal.cn/datasheet.jsp?productId=72026&images=1&size=A4

Antibodies for flow cytometry
CD16/32 BioLegend #101302, clone: 93, 1:1000
https://www.biolegend.com/en-us/products/purified-anti-mouse-cd16-32-antibody-190
CD45-PerCP/Cy5.5 BioLegend #103131 clone: 30-F11 1:1000
https://www.biolegend.com/en-us/products/percp-cyanine5-5-anti-mouse-cd45-antibody-4264
CD45-AF700 BD Biosciences #560510 clone: 30-F11 1:800
https://www.bdbiosciences.com/content/bdb/paths/generate-tds-document.us.560510.pdf
CD31-PerCP/Cy5.5 BioLegend #102419 clone:390 1:300
https://www.biolegend.com/en-us/products/percp-cyanine5-5-anti-mouse-cd31-antibody-6668
F4/80-PE Cell Signaling #64763 1:80
https://www.cellsignal.cn/datasheet.jsp?productId=64763&images=1&size=A4
CD11c-AF647 BioLegend #117314 clone: N418 1:100
https://www.biolegend.com/en-us/products/alexa-fluor-647-anti-mouse-cd11c-antibody-2703
CD206-PE Cy7 BioLegend #141720 clone: C068C2 1:100

https://www.biolegend.com/en-us/products/pe-cyanine7-anti-mouse-cd206-mmr-antibody-8631
CD26 (DPP4)-APC BioLegend #137807 clone: H194-112 1:100
https://www.biolegend.com/en-us/products/apc-anti-mouse-cd26-dpp-4-antibody-6947
CD54 (ICAM1)-PE/Cy7 BioLegend #116121 clone: YN1/1.7.4 1:100
https://www.biolegend.com/en-us/products/pe-cyanine7-anti-mouse-cd54-antibody-14759
Anti-F4/80-Biotin Miltenyi Biotech. #130-116-514 1:100
https://www.miltenyibiotec.com/CN-en/products/f4-80-antibody-anti-mouse-reafinity-rea126.html#biotin:30-ug-in-200-ul
Antibodies for immunohistochemistry
Rabbit anti-F4/80 #70076 Cell Signaling 1:200
https://www.cellsignal.cn/datasheet.jsp?productId=70076&images=0&size=A4

Antibodies for immunofluorescence
Rabbit anti-αSMA Cell Signaling #19245 1:5000
https://www.cellsignal.cn/datasheet.jsp?productId=19245&images=1&size=A4
Rabbit anti-CD26 (DPP4)-APC BioLegend #137807 clone: H194-112 1:100
https://www.biolegend.com/en-us/products/apc-anti-mouse-cd26-dpp-4-antibody-6947
Rabbit anti-Ki67-eFluor 660 eBioscience #50-5698-82 1:200
https://www.thermofisher.cn/cn/zh/antibody/product/Ki-67-Antibody-clone-SolA15-Monoclonal/50-5698-82
Rabbit anti-p-SMAD2 (S465/S467) Cell Signaling #18338, 1:100
https://www.cellsignal.cn/datasheet.jsp?productId=18338&images=1&size=A4
Rabbit anti-Col1a1 Cell Signaling #72026 1:100
https://www.cellsignal.cn/datasheet.jsp?productId=72026&images=1&size=A4

Eukaryotic cell lines

Policy information about cell lines

Cell line source(s)	HEK 293T (CRL-3216) was obtained from American Type Culture Collection (ATCC).
Authentication	None of the cell lines have been authenticated.
Mycoplasma contamination	Frozen batch used for experiment was tested for mycoplasma and resulted negative.
Commonly misidentified lines (See ICLAC register)	No commonly misidentified cell lines were used in the study.

Animals and other organisms

Policy information about studies involving animals; ARRIVE guidelines recommended for reporting animal research

Laboratory animals	Lats1f/f (024941, C57BL/6-129-CD1), Lats2f/f (027934, C57BL/6-129), Yap1f/f (027929, C57BL/6-129), ob/+ (000632), ROSAmT/mG (007676), Rosa26-floxed STOP-Cas9-EGFP mice (026179), EIIaCre (003724), AdipoqCre (028020) and AdipoqCreERT2 (025124) mice were obtained from The Jackson Laboratories. Tazf/f mice (C57BL/6-129) were made as below: a plasmid donor, containing two 34-bp loxP sites in homologous arms flanking the exon 2 of Wwtr1 gene, was designed to generate a Wwtr1 floxed allele by homologous recombination. Rosa26-floxed STOP-Cas9-EGFP mice were crossed with either EIIaCre or AdipoqCre to generate a mouse line with constitutive expression of CAS9 in all tissues or in adipocytes. Unless noted otherwise, mice are on C57BL/6 background. The sex and age of all the strains were indicated in the legends. Mice were housed in temperature (22 ± 1°C)- and humidity (60 ± 10%)-controlled rooms under a 12-h light-dark cycle, provided with chow diet and water ad libitum, except for fasting experiments.
Wild animals	This study did not involve wild animals.
Field-collected samples	This study did not involve field-collected samples.
Ethics oversight	All animal procedures were performed in compliance with protocols approved by the Institutional Animal Care and Use Committee of Peking University and conformed to the Guide for the Care and Use of Laboratory Animals.

Note that full information on the approval of the study protocol must also be provided in the manuscript.

Flow Cytometry

Plots

Confirm that:

- [x] The axis labels state the marker and fluorochrome used (e.g. CD4-FITC).
- [x] The axis scales are clearly visible. Include numbers along axes only for bottom left plot of group (a 'group' is an analysis of identical markers).
- [x] All plots are contour plots with outliers or pseudocolor plots.
- [x] A numerical value for number of cells or percentage (with statistics) is provided.

Methodology

Sample preparation	Inguinal scWAT was minced with scissors and digested with Collagenase Type I (180 U/ml, Worthington, LS004216) in SVF buffer [1.1 mM $CaCl_2$, 2.7 mM KCl, 118 mM NaCl, 0.5 mM $MgCl_2$, 0.4 mM NaH_2PO_4, 20 mM HEPES, 5.5 mM Glucose, 1% BSA (fatty-acid free)] at 37°C with agitation at 100 rpm for 50 min. The digested cell suspension was centrifuged at 520 × g for 5 min, resuspended in SVF buffer and passed through a 40-µm strainer. Floated cells were collected as adipocytes, and pelleted cells were resuspended in red blood cell lysis buffer (0.15 M NH_4Cl, 10 mM $NaHCO_3$, 1.1 mM EDTA) for 5 min at RT and then quenched in SVF buffer. Next, cells were collected by centrifugation at 520 × g for 5 min and recovered in FACS buffer (PBS containing 2% FBS and 1 mM EDTA) for further staining.
Instrument	Beckman Coulter CytoFLEX S
Software	CytExpert v2.3.0.84 , Flowjo v10.
Cell population abundance	The abundance of cell populations was presented in the graphs in Figure 5g, Extended Data Figure 8a and Extended Data Figure 9l.
Gating strategy	We first gated the singlets by FSC-A and FSC-H, and exclude dead cells. Specific cell populations were determined by markers listed below.<br>Macrophages: CD45+/F4/80+; M1 macrophages: CD45+/F4/80+/CD11c+/CD206-; M2 macrophages: CD45+/F4/80+/CD11c-/CD206+. GFP+DPP4+ cells: CD45-CD31-GFP+DPP4+ ; DPP4+ cells: CD45-CD31-DPP4+; DPP4- cells: CD45-CD31-DPP4- cells; DPP4+ ICAM1-cells: CD45-CD31-DPP4+ICAM1-; DPP4- ICAM1+ cells: CD45-CD31-DPP4-ICAM1+.

☒ Tick this box to confirm that a figure exemplifying the gating strategy is provided in the Supplementary Information.
